# Pharmacophore-based virtual screening, molecular docking, and molecular dynamics investigation for the identification of novel, marine aromatase inhibitors

**DOI:** 10.1186/s13065-024-01350-9

**Published:** 2024-11-26

**Authors:** Mohamed A. Kotb, Islam Ahmed Abdelmawgood, Ibrahim M. Ibrahim

**Affiliations:** 1 1Department of Zoology, Faculty of Science, Cairo University, Giza, Egypt; 2 2Department of Biophysics, Faculty of Science, Cairo University, Giza, Egypt

**Keywords:** Molecular docking, Pharmacophore, Virtual screening, Molecular dynamics, Aromatase, Aromatase inhibitors

## Abstract

Breast cancer remains a leading cause of mortality among women worldwide. Our current research focuses on identifying effective therapeutic agents by targeting the human aromatase enzyme. Aromatase inhibitors (AIs) have been effective in treating postmenopausal breast cancer but face challenges such as drug resistance and long-term side effects like cognitive decline and osteoporosis. Natural products, especially from marine organisms, are emerging as potential sources for new drug candidates due to their structural diversity and pharmacological properties. This study aims to discover marine natural products capable of inhibiting human aromatase by combining ligand-based and structure-based pharmacophore models for virtual screening against the Comprehensive Marine Natural Products Database. From the initial virtual screening of more than 31,000 compounds, 1,385 marine natural products were identified as possible candidates. Following initial molecular docking analysis, only four compounds managed to pass the criteria this research has introduced to confirm strong binding affinity to aromatase. All four compounds yielded acceptable binding affinities, with CMPND 27987 having the highest −10.1 kcal/mol. All four hits were subjected to molecular dynamics, and CMPND 27987 was further confirmed to be the most stable at the protein’s active site, with an MM-GBSA free binding energy of −27.75 kcal/mol. Our in silico studies indicate that CMPND 27987 interacts effectively within the binding site of the human aromatase, maintaining high affinity and stability. Based on these findings, we propose that CMPND 27987 could hold significant potential for further lead optimization and drug development.

**Supplementary Information:**

The online version contains supplementary material available at 10.1186/s13065-024-01350-9.

## Introduction

**Fig. 1:**
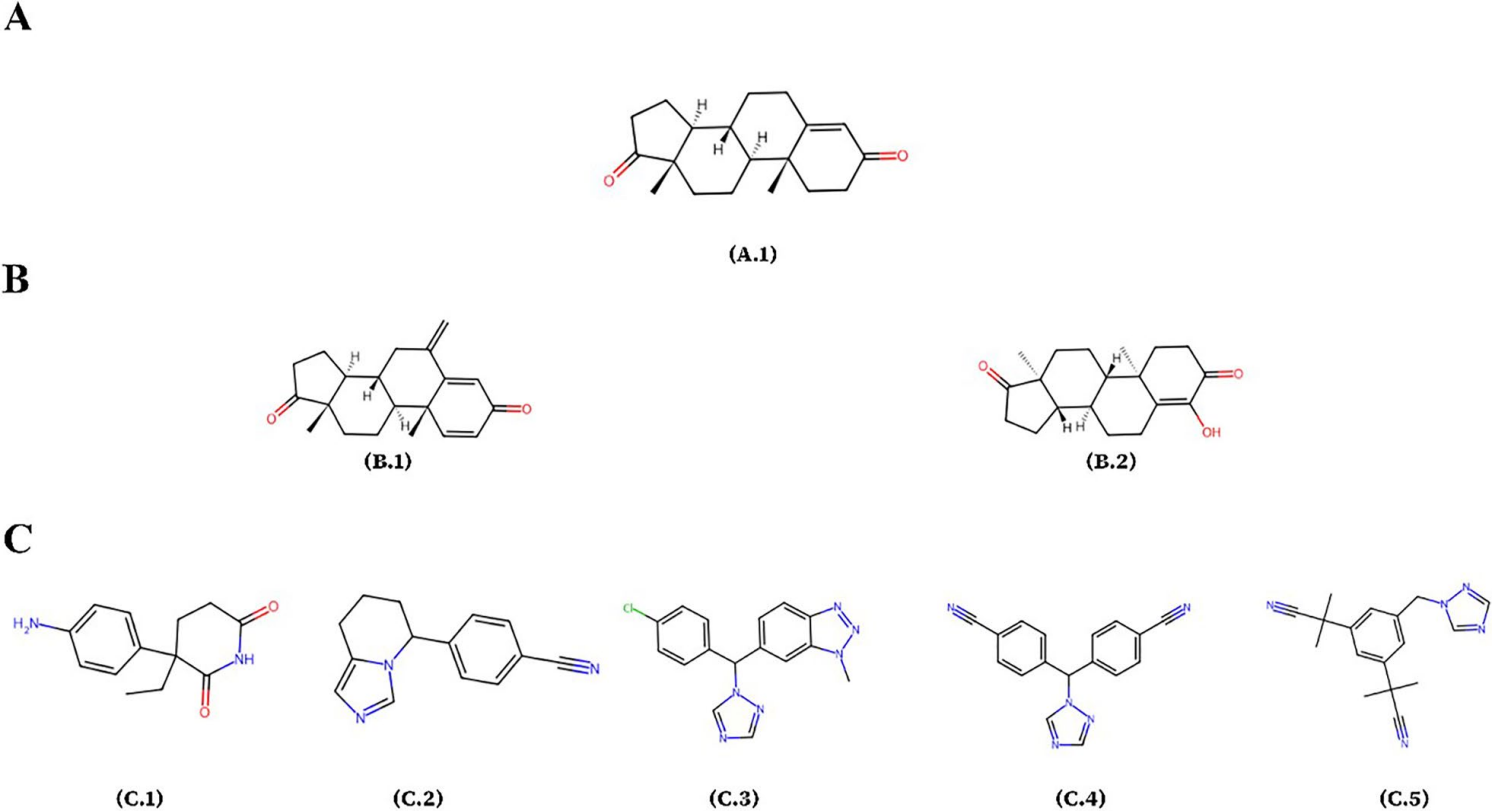
The figure shows the human aromatase ligands, including (**A**) the co-crystalized ligand and androgen precursor androstenedione (**A1**), (**B**) type I inhibitors such as exemestane (third generation) (**B2**) and formestane (second generation) (**B2**), and (**C**), type II inhibitors aminoglutethimide (first generation) (**C1**), fadrozole (third generation) (**C2**), and the triazoles: vorozole (third generation) (**C3**),  letrozole (third generation) (**C4**), and anastrozole (third generation (**C5**)

Female breast cancer was the main cause of global cancer incidence in 2020, totaling 2.3 million new cases and comprising 11.7% of the overall cancer burden. In terms of fatalities, it contributed to 685,000 deaths, constituting 1 in 6 cancer-related mortalities [1]. A sizable proportion of breast cancers are estrogen-receptor positive and thus are categorized as hormone-dependent [2]. The dysregulated interplay between estrogens and estrogen receptors (ERs) within the tumor triggers mitogenic signals that are strongly connected to breast tumor proliferation and invasion [3]. In contrast to premenopausal women, where the primary source of estrogens is ovarian synthesis, postmenopausal women, comprising the majority of breast cancer patients, possess estrogens that are either produced in nonovarian tissues or synthesized peripherally through the human aromatase enzyme [4]. The human aromatase enzyme CYP 450 (CYP19A1), a member of the cytochrome P450 family, is the translation product of the CYP19A1 gene on chromosome 15. It catalyzes the rate-limiting and conclusive step of extra-ovarian aromatization of the A ring of androgen precursors, such as androstenedione (ASD) (Fig. 1A), to synthesize estrogens [5]. Two primary pharmacological strategies have been employed in the management of breast cancer. The first involves endocrine therapy, which acts through ER antagonism, while the second uses aromatase inhibitors (AIs) that disrupt the exogenous estrogen synthesis through aromatase inhibition [6].

Breast cancer treatment has traditionally used endocrine therapy. It utilizes selective estrogen receptor modulators (SERMs), such as Tamoxifen, to impede estrogen binding to ERs. SERMs, acting as agonists or exerting estrogenic effects in other tissues, are associated with several side effects, including an elevated risk of thromboembolism and endometrial carcinoma [7].

AIs are classified into Type I (steroidal) and Type II (non-steroidal) inhibitors. Type I inhibitors, such as formestane (second generation) and exemestane (third generation) (Fig. 1B), mimic the structure of the natural steroidal substrate, binding covalently and irreversibly to CYP19A1. Type II inhibitors include aminoglutethimide (first generation), fadrozole (third generation), and the triazoles: vorozole (third generation), anastrozole (third generation), and letrozole (third generation) (Fig. 1c). These compounds, especially the latter three, reversibly coordinate the iron atom in the enzyme's heme group through a heterocyclic nitrogen lone pair [8].

The inhibition of human aromatase proves to be associated with fewer side effects compared to endocrine therapy, as it lacks additional estrogenic effects and has consistently demonstrated effectiveness in regulating the regression of breast tumors that depend on estrogenic stimulation for their development and growth. [9]. These findings resulted in the further development of AIs, which marked a breakthrough in breast cancer therapy [10].

AIs have been useful in postmenopausal breast cancer treatment, but novel drugs are needed to avoid drug resistance, especially in metastatic breast cancer, and reduce toxicity and side effects from long-term use [11]. Prolonged AI use can change patients' lipid profiles and bone mineral density, cause osteoporosis, and increase the risk of musculoskeletal disorders. It also affects cognition by reducing estrogen's protective effects on age-related memory loss. [12].

Due to their structural and pharmacological diversity, natural compounds have long been used in drug discovery as a source of new lead candidates. [13]. As with terrestrial plants, fungi, and bacteria, marine organisms are under investigation for their pharmaceutical potential [14]. Recently discovered marine natural products (MNPs) have led to the development of anti-inflammatory, antiviral, antibacterial, and anticancer drugs. [15–17]. In the context of AIs, natural products have the potential to facilitate the transition from their current clinical use as chemotherapeutic agents to future applications in breast cancer chemoprevention [18].

Molecular docking, molecular dynamics, ligand-based and structure-based pharmacophore modeling, virtual screening, and other computer-aided drug design methods have become essential for drug design [19, 20]. These techniques are central for selecting promising lead candidates for biological testing [21].

Our study aims to identify marine natural product(s) capable of binding to the human aromatase enzyme at the active site. To accomplish this, a ligand-based pharmacophore model derived from a series of novel, non-steroidal AIs was merged with a docking-assisted structural-based pharmacophore model. The resulting pharmacophore was screened against the Comprehensive Marine Natural Products Database (CMNPD), a manually curated open-access knowledge base specifically designed for marine natural products research [22] to identify natural AIs. Further confirmation was conducted through the employment of molecular docking and molecular dynamics studies.

## Methods

### Pharmacophore modeling and virtual screening.

Third-generation, non-steroidal AIs have a nitrogen-containing heterocyclic group that directly interacts with Fe (II) of the heme group, preventing aromatization. In the search for lead candidates, azaheterocycle pharmacophore scaffolds are becoming a more reliable option [23].

A comprehensive literature review identified a series of non-steroidal AIs with the azole group at the 3rd position in a 2-phenyl indole scaffold (Table 1). According to Kang et al., the indole moiety fits in the catalytic cleft of the human aromatase, while the azaheterocyclic moiety chelates the heme group's iron atom. Compound 6 with a triazole ring showed the highest anti-aromatase activity, surpassing letrozole (0.0495 μM) with an IC50 value of 0.0141 μM [24].

**Table 1:** The structure and cytotoxicity of 2-phenyl indole-based aromatase inhibitors [24]

Compound	Structure	IC_50_ (µM)	Compound	Structure	IC_50_ (µM)
Compound 6		0.0141	Compound 10		0.1965
Compound 19		0.0323	Compound 12		0.2075
Compound 15		0.0361	Compound 5		0.2106
Compound 9		0.0512	Compound 13		0.2174
Compound 3		0.0544	Compound 20		0.2376
Compound 17		0.0583	Compound 2		0.2608
Compound 14		0.0829	Compound 7		0.2768
Compound 18		0.0969	Compound 8		0.6774
Compound 16		0.1087	Compound 4		1.0580

This series was used to start a ligand-based pharmacophore model. A docking-assisted structural-based model (supplementary material) was built using Compound 6, which had the highest biological efficacy

#### Ligand-based pharmacophore modeling

The series of 18 compounds in Table 1 was sketched in MarvinSketch and saved as SDF files. The Merck Molecular Force Field (MMFF94) was used to minimize the three-dimensional coordinates of these files using an in-house Python script.

LigandScout 4.3 divided the series into 14 training compounds and 4 test compounds at random. Then, the program's Conformer Generation Settings were used to perform a conformational analysis with the Best Settings and 100 conformers.

Pharmacophore fit and atom overlap scoring function created the ligand-based pharmacophore. The merged Pharmacophore type had 2 omitted features.

#### Docking-assisted structural-based pharmacophore modeling.

Compound 6 was used to build a structural-based pharmacophore model using molecular docking. This method was chosen because non-steroidal aromatase inhibitor binding interactions are not crystallographically documented.

The interaction of Type II AIs, including letrozole, with aromatase leads to a notable bathochromic shift in the Soret UV band when compared to Type I inhibitors [25, 26]. This shift may be caused by the coordination of the heme iron with a heteroatom (N, S, O, S-), such as letrozole's triazolic ring nitrogen. In their molecular docking study, Galeazzi and Massaccesi found two letrozole poses with CYP19A1. The first pose had higher binding energy and had the triazole ring away from the heme center, while the second had the azaheterocyclic ring toward it. Despite having lower binding energy, the second pose matched UV-absorption spectrum data, which the pair confirmed through molecular dynamics studies [27]. The supplementary material details the method utilized for selecting the appropriate pose for molecular docking-assisted pharmacophore modeling.

The X-ray diffraction crystallographic structure of the human aromatase enzyme (PDB ID: 3EQM) with a resolution of 2.90 Å was obtained from the Protein Data Bank for molecular docking Molecular visualization software PyMol removed the co-crystallized ligand ASD and added hydrogens [28]. Catalytic water molecules were maintained as previous research has pointed out their involvement in the hydroxylation mechanism of aromatization in CYP19A1 [29]. The active site was visualized using MGL Tools [30]. Box size (16, 20, 16) and box center (85.1, 51.016, 43.076) were assigned using 1 Å spacing. Finally, the AutoDock Vina program performed molecular docking with 100 exhaustiveness. [31].

Utilizing the same settings in LigandScout that were used to establish the ligand-based pharmacophore model. Our group created a structural-based pharmacophore model.

#### Merged pharmacophore generation

Using the alignment module of LigandScout, the two previous models were aligned based on their features, and the common pharmacophoric attributes were merged to create a unified model.

#### Benchmarking database, selecting the best model, and virtual screening

A database of 40 highly active aromatase inhibitors of various scaffolds sourced from the literature was used to generate 1,509 decoys using the DecoyFinder program [32] from the drug-like subset of the ZINC15 database [33]. To improve the decoys' reliability, 815 compounds were selected from the program's output. In AutodockVina docking with aromatase, this subset represented decoys with the lowest binding energy. The three models were benchmarked using LigandScout decoys and a pharmacophore-fit scoring function matching all query features and a maximum of 2 omitted features with volume exclusion. After that, ROC analysis determined each pharmacophore model's selectivity and specificity.

The CMNPD database was imported onto LigandScout, and pharmacophore-based virtual screening was conducted according to the aforementioned parameters.

### Molecular docking study

The pharmacophore-based virtual screening hits underwent molecular docking analysis using AutoDock Vina. The same docking parameters mentioned previously were employed. Compounds that had a docking score higher than −7 kcal/mol, a pharmacophore fit score higher than 70, and an azaheterocyclic ring were filtered to be used in molecular dynamics studies. Clustering analysis methodology is provided in the supplementary material.

### Molecular dynamics simulation

Various compounds, including Androstenedione (co-crystal ligand), CMNPD7905, CMNPD7907, CMNPD11121, CMNPD27987, Formestane (second generation AI), and Letrozole (third generation AI), were used in unbiased molecular dynamics (MD) simulation utilizing GROMACS 2021 software, with a simulation duration of 200 ns [34]. To prepare the necessary input files, the solution builder module of the CHARMM-GUI server was utilized [35–38]. Each complex was placed in a 9.4-nm cubic box in solvent. The transferable intermolecular potential 3 points (TIP3P) water model and a padding region 1 nm beyond the farthest atom were used in the solvation process. For system neutrality, 0.154 M NaCl ions were added. The CHARMM36m force field determined CYP450 protein amino acid parameters, TIP3P water model parameters, and neutralizing ion parameters. CHARMM general force field (CGenFF) parameterized molecules.

Periodic boundary conditions (PBC) were used in all three dimensions during simulation. Prevention of atomic collisions involved potential energy minimization. After 100,000 minimization steps or when the maximum force applied to any atom dropped below 100 kJ/(mol.nm), the minimization process converged. Two equilibration stages established thermal and pressure equilibrium in the systems. The NVT ensemble set an average temperature of 310 K using the Velocity Rescale method during initial equilibration. In the next stage, the NPT ensemble used the Berendsen barostat and velocity rescaling to maintain 1 atm atmospheric pressure and 310 K average temperature [39]. Throughout the 200 ns production run, the NPT ensemble was employed, with temperature control achieved using the Nose–Hoover thermostat and pressure control maintained by the Parrinello-Rahman barostat. The temperature was maintained at 310 Kelvin, while the pressure was kept at 1 atmosphere [40]. To impose constraints on the lengths of hydrogen-bonded atoms, the LINear Constraint Solver (LINCS) algorithm was utilized [41]. Electrostatic calculations were performed using the Particle Mesh Ewald (PME) method with a threshold of 1.2 nm [42]. The Newtonian equations of motion were numerically integrated using leap-frog integration with a time step of 1 femtosecond during equilibration and 2 during production. At 0.1 ns intervals, 2,000 frames were recorded during the simulation.

Following the repositioning of the protein within the periodic box to restore its structural integrity using the gmx trjconv tool, a comprehensive examination of the trajectory was conducted using VMD TK scripts [43]. The Root Mean Square Deviation (RMSD) for the CYP450 protein and each compound was calculated using several methods. Other structural characteristics such as Root Mean Square Fluctuation (RMSF), Radius of Gyration (RoG), Solvent Accessible Surface Area (SASA), hydrogen bond number between compounds and CYP450, and ligand distance from protein center of mass were also examined. To study ligand-amino acid interactions, each trajectory frame was carefully examined. This was done using the Protein–Ligand Interaction Fingerprints (ProLIF) Python program to identify interacting amino acids and assess their stability importance [44].

#### Binding free energy calculation using MM-GBSA

The gmx_MMPBSA program used MM-GBSA to calculate the ligand's binding energy. Additionally, a decomposition analysis assessed the binding contribution of each amino acid within a 1-nm radius of the ligand. [45, 46]. The selected parameters encompassed an ionic strength of 0.154 M and a solvation technique (igb) value of 5. The internal dielectric constant was set at 1.0, while the external dielectric constant was set at 78.5. Mathematically, the MM-GBSA method can be represented by Eq. 1.

1 $$\Delta {\text{G = < Gcomplex - (Greceptor + Gligand) > }}$$

where <  > represents the average of the enclosed free energies of complex, receptor, and ligand over the frames used in the calculation. In our approach, we used the whole trajectory (a total of 2000 frames). Different energy terms can be calculated according to Eqs. 2, 3, 4, 5, 6 as follows:

2 $$\Delta {\text{Gbinding = }}\Delta {\text{H - T}}\Delta {\text{S}}$$

3 $$\Delta {\text{H = }}\Delta {\text{Egas + }}\Delta {\text{Esol}}$$

4 $$\Delta {\text{Egas = }}\Delta {\text{Eele + }}\Delta {\text{EvdW}}$$

5 $$\Delta {\text{Esolv = EGB + ESA}}$$

6 $${\text{ESA = }}\gamma {\text{.SASA}}$$

where:

∆H is the enthalpy which can be calculated from gas-phase energy (E_gas_) and solvation-free energy (E_sol_). -T∆S is the entropy contribution to the free binding energy. E_gas_ is composed of electrostatic and van der Waals terms; E_ele_, E_vdW_, respectively. E_sol_ can be calculated from the polar solvation energy (E_GB_) and nonpolar solvation energy (E_SA_) which is estimated from the solvent-accessible surface area [47, 48].

## Results

### Pharmacophore modeling and virtual screening.

#### Ligand-based, structural-based, and merged pharmacophore modeling

**Fig. 2:**
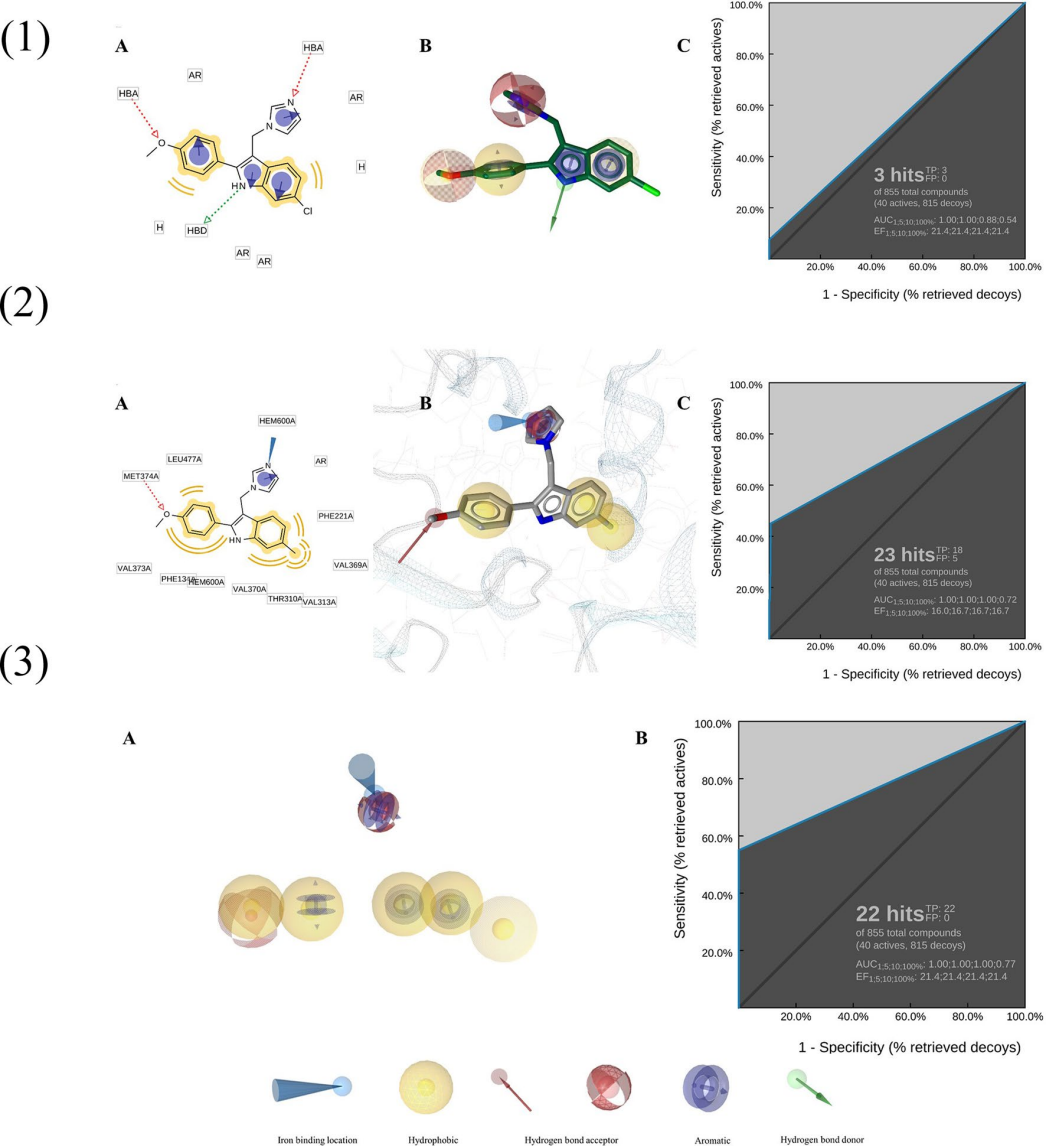
Pharmacophore models. (**1**) Ligand-based pharmacophore model. (1.A) Two-dimensional representation of ligand-based pharmacophore mode showcasing important functional groups. (1.B) Three-dimensional representation of ligand-based pharmacophore mode showcasing important functional groups. (1.C) ROC analysis for ligand-based model. (**2**) Structural-based pharmacophore model. (2.A) Two-dimensional representation of Structural-based pharmacophore mode showcasing important functional groups. (2.B) Three-dimensional representation of structure-based pharmacophore mode showcasing important functional groups. (2.C) ROC analysis for the structure-based model. (**3**) Merged pharmacophore model. (3.A) Two-dimensional representation of merged model. (3.B) Three-dimensional representation of merged pharmacophore moded. (3.C) ROC analysis for merged model

LigandScout selected the top-scoring ligand-based pharmacophore model (0.94) from 10 models. Compound 6 scored 92.5 pharmacophore-fit. In this model, the heterocyclic group and para 2-phenyl position had hydrogen bond acceptors, and the 2-phenyl position and indole's benzene ring had hydrophobic moieties. The pyrrole ring of indole had an aromatic moiety with a hydrogen bond donor at nitrogen (Fig. 2.1). A benchmarking study using 40 active compounds and 815 decoys verified the model's virtual screening reliability. ROC analysis showed AUC of 0.54, EF of 21.4, and pAUC values of 1.00, 1.00, and 0.88 at 1%, 5%, and 10% of the screened database (Fig. 2C).

Supplemental material contains docking-assisted structural-based pharmacophore model cluster analysis data. The structure-based pharmacophore model found seven features, including an iron-binding site, aromatic ring, and hydrogen bond acceptor at Compound 6's azaheterocyclic ring.The model showcased possible hydrogen bonding at the Met374 position. Hydrophobic interactions were observed with Phe134, Val370, and other binding site residues. ROC analysis yielded 23 hits (18 actives, 5 decoys), 0.72 AUC, 16.7 EF, and 1.00 pAUC at 1%, 5%, and 10% (Fig. 2.2C).

A merged model with 22 true positives and no decoys retained key shared and unique pharmacophoric elements from both approaches. AUC was 0.77, EF 21.4, and pAUC 1.00 at 1%, 5%, and 10% (Fig. 2.3A).

#### Pharmacophore-based virtual screening.

The open-access CMNPD database supports marine natural products research [22]. It covers many chemical entities' physicochemical and pharmacokinetic properties. Standardized biological activity data, systematic taxonomy, source organism geographical distribution, and literature citations are also available in the database. About 31,000 compounds from 3,400 marine organisms make up CMNPD. After importing the database into LigandScout, pharmacophore-based virtual screening was performed using the method parameters. The screening yielded 1,385 hits with 53.32–81.48 pharmacophore-fit scores.

### Molecular docking study

#### Molecular docking using AutoDockVina

The parameters for molecular docking were applied to all virtual screening hits using AutoDock Vina. Four compounds—CMNPD27987, CMNPD11121, CMNPD7905, and CMNPD7907 (Table 2)—met the selection criteria with docking scores above −7 kcal/mol, pharmacophore fit scores above 70, and containing azaheterocyclic rings. These four compounds were included in subsequent molecular dynamics studies. The study included androstenedione (co-crystal ligand), formestane (type I AI), and letrozole (type II AI and reference drug) as controls.

**Table 2:** Candidate and reference drug names, docking binding energies, pharmacophore-fit scores, and chemical structures

Name	Binding energy (Kcal/mol)	Pharmacophore-Fit Score		Structure
Androstenedione (ASD)	−14	*NA*		
Formestane	−13.1	*NA*		
Letrozole	−7.1	66.82		
Compound 27,987	−10.1	72.18		
Compound 11,121	−10	71.81		
Compound 7905	−8.6	76.18		
Compound 7907	−8.6	76.19		

The bioactive pose candidate was identified manually, like the structure-based pharmacophore model. This required hierarchical cluster analysis of poses generated over 100 iterations, calculation of RMSD between cluster representatives' azaheterocyclic rings and letrozole's, and measurement of the distance between the center and the heme group's Fe atom. Molecular docking cluster analysis data is in the supplementary material

#### Structural analysis of protein–ligand interaction profiles.

**Fig. 3:**
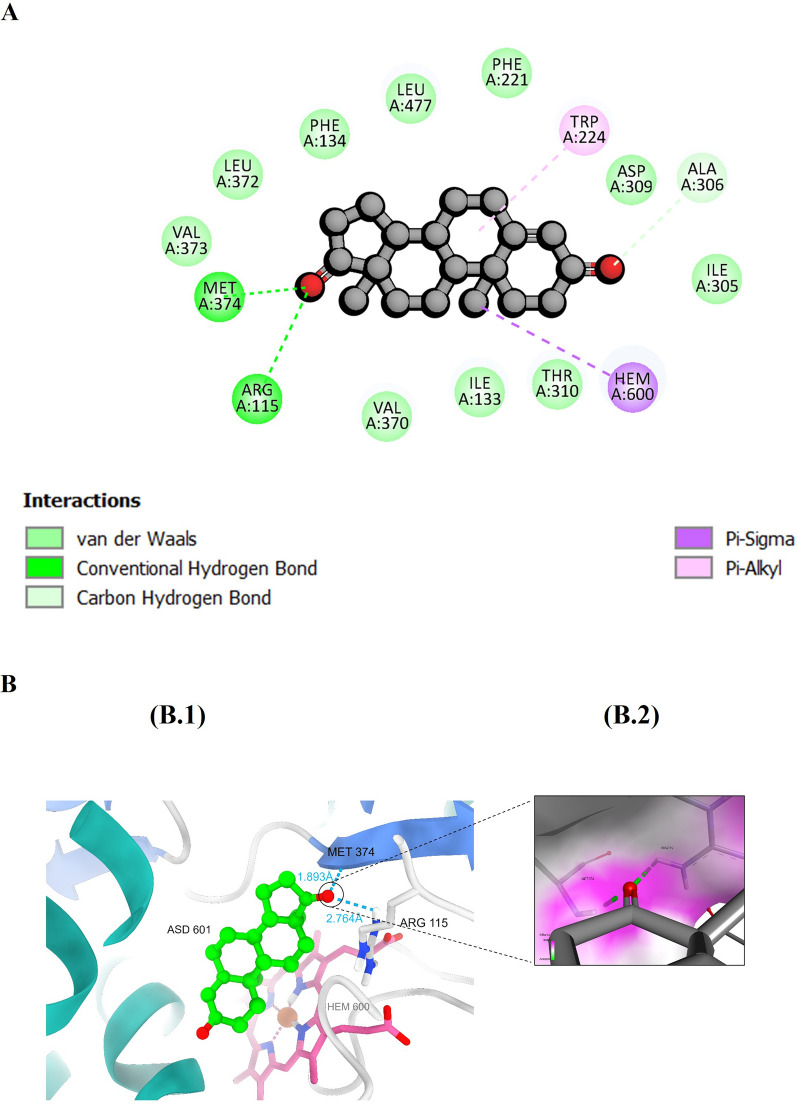
The figure shows (**A**) A two-dimensional representation of the interaction profile between ASD and the human aromatase enzyme. **B** demonstrates hydrogen bond interactions within the binding site, with (B.1) showcasing the overall interactions and (B.2) providing a closer view of the hydrogen bond interaction with a surface representation

Analysis of the protein–ligand interaction profile of the co-crystal ligand androstenedione revealed the formation of two hydrogen bonding interactions with amino acid residues Arg115 and Met374. Several carbon-hydrogen bonds with Arg115, Ala306, Val373, and Met374 were also observed. Two hydrophobic interactions: a Pi-sigma interaction with the heme moiety and a Pi-alkyl interaction with Trp224. A number of van de Waals interactions with amino acid residues of the binding pocket include Phe221, Leu477, Phe134, Leu372, Val373, Val370, Ile133, Thr310, Ile305, and Asp309 (Fig. 3).

**Fig. 4:**
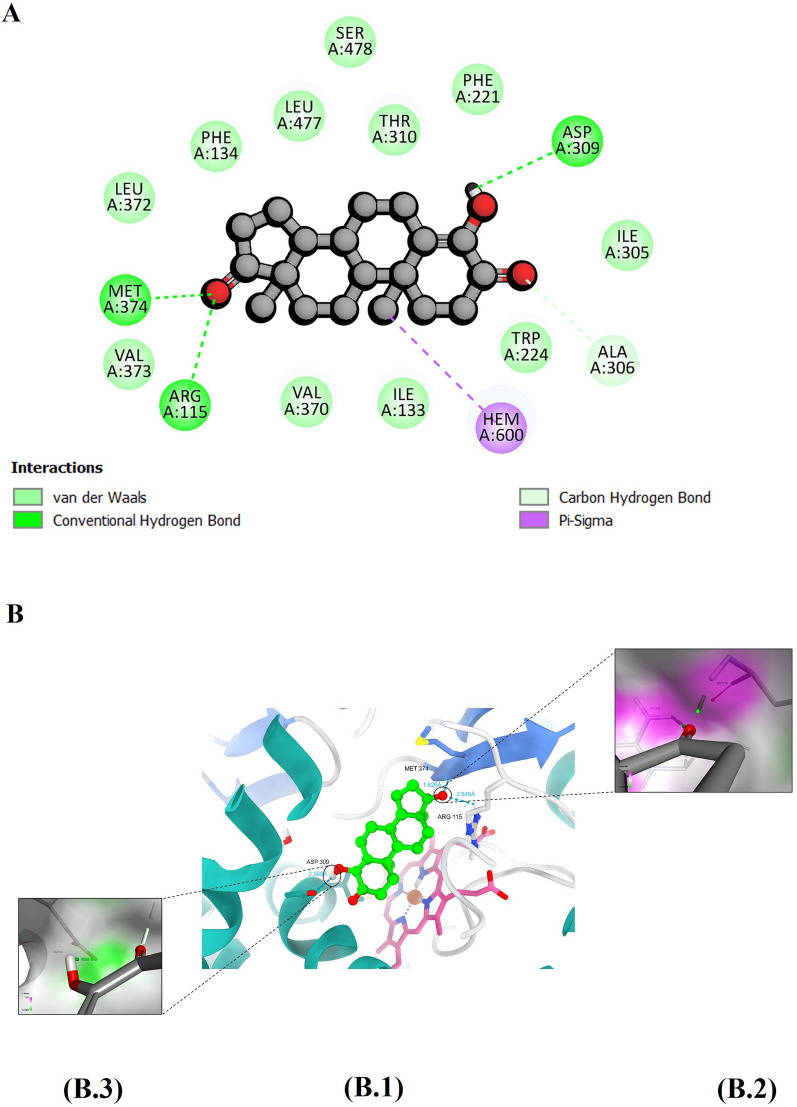
The figure demonstrates (**A**) Depiction of a two-dimensional representation illustrating the interaction profile between formestane and the human aromatase enzyme. **B** illustrates hydrogen bond interactions within the binding site, with (B1) presenting an overview of the interactions and (B 2 and 3) offering a close-up view of the hydrogen bond interaction along with a surface representation

Formestane, a second-generation, steroidal AI, has a structure similar to the co-crystalized ligand, wherein the hydrogen at position 4 is replaced by a hydroxy group. In terms of hydrogen bonds, it forms similar interactions with amino acid residues Arg115 and Met375; however, it shows an additional hydrogen bonding interaction with Asp309, in which the 4-hydroxy acts as a hydrogen bond donor (Fig. 4B3). Formestane has a total of 11 van der Waal interactions with the following amino acids: Trp224, Ile305, Asp309, Ser478, Phe221, Thr310, Phe134, Leu372, Val373, Val370, and Ile133 (Fig. 4A).

**Fig. 5:**
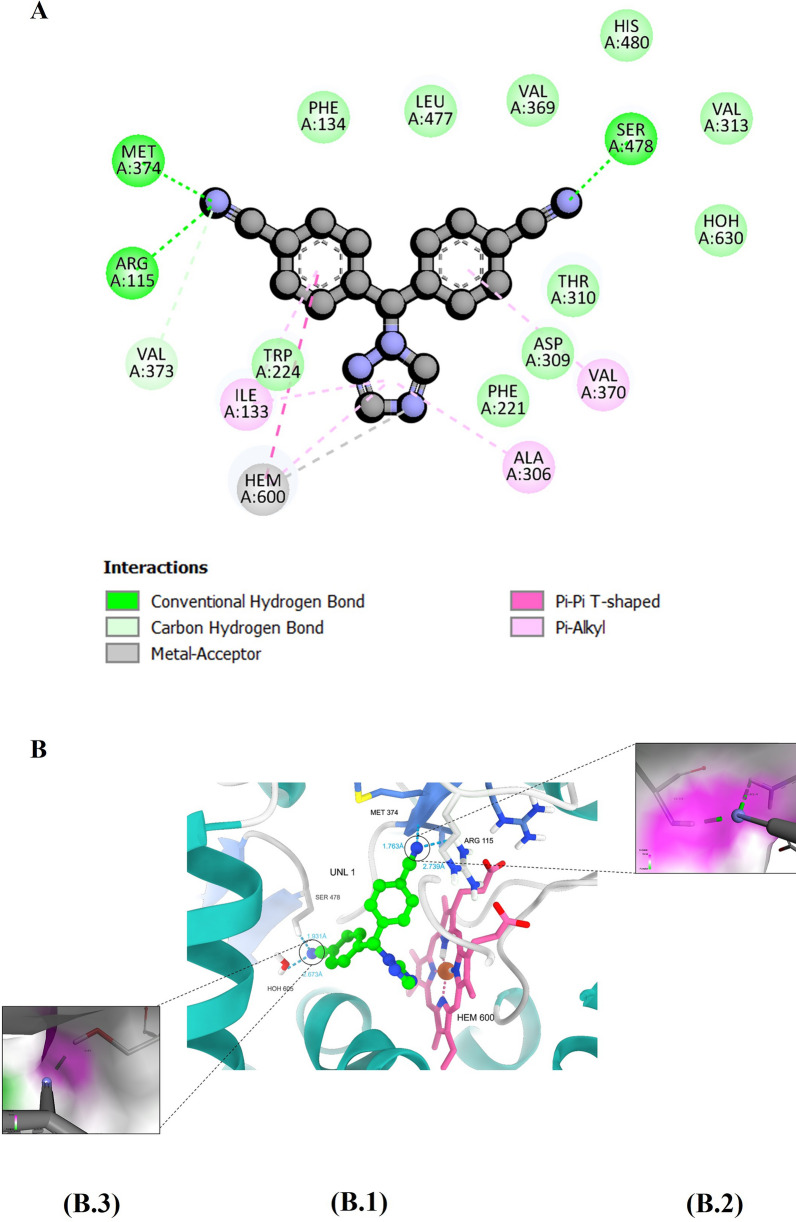
**A** A depiction of a two-dimensional representation illustrating the interaction profile between formestane and the human aromatase enzyme. **B** illustrates hydrogen bond interactions within the binding site, with (B1) presenting an overview of the interactions and (B 2 and 3) offering a close-up view of the hydrogen bond interaction along with a surface representation

The binding interactions of letrozole, a third-generation non-steroidal AI and an in-market drug, had the highest number of interactions when compared to androstenedione and formestane. Letrozole formed three strong conventional hydrogen bonding interactions with amino acid residues Arg115, Met374, and Ser478. Amino acid residues His480, Val369, Ser478, Leu477, Phe134, Val373, Trp224, Phe221, Asp309, Thr310, Val313, and HOH630 participated in van der Waal interactions with letrozole (Fig. 5A).

**Fig. 6:**
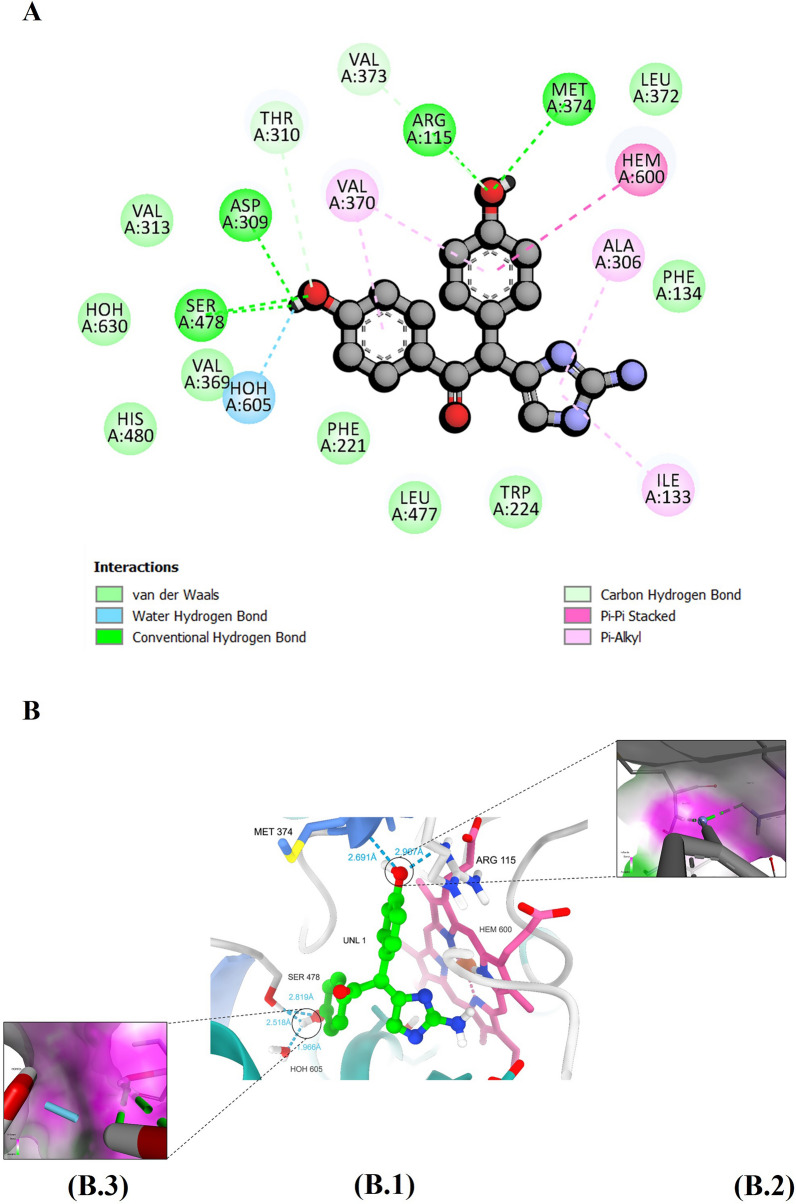
**A** A two-dimensional representation illustrating the interaction profile between letrozole and the human aromatase enzyme. **B** illustrates hydrogen bond interactions within the binding site, with (D1) presenting an overview of the interactions and (B 1 and 2) providing a close-up view of the hydrogen bond interaction along with a surface representation

Similar to letrozole, the first hit, CMNPD27987, shows hydrogen bonding interactions with Arg115, Met374, and Ser478. However, it forms an additional hydrogen bond with the water molecule HOH605 of the binding site (Fig. 6B) Van der Waal interactions between CMNPD27987 and CYP19A1 include Leu372, Arg115, Phe134, Val313, His480, Val369, Phe221, Asp309, Leu477, and Trp224 (Fig. 6A).

**Fig. 7:**
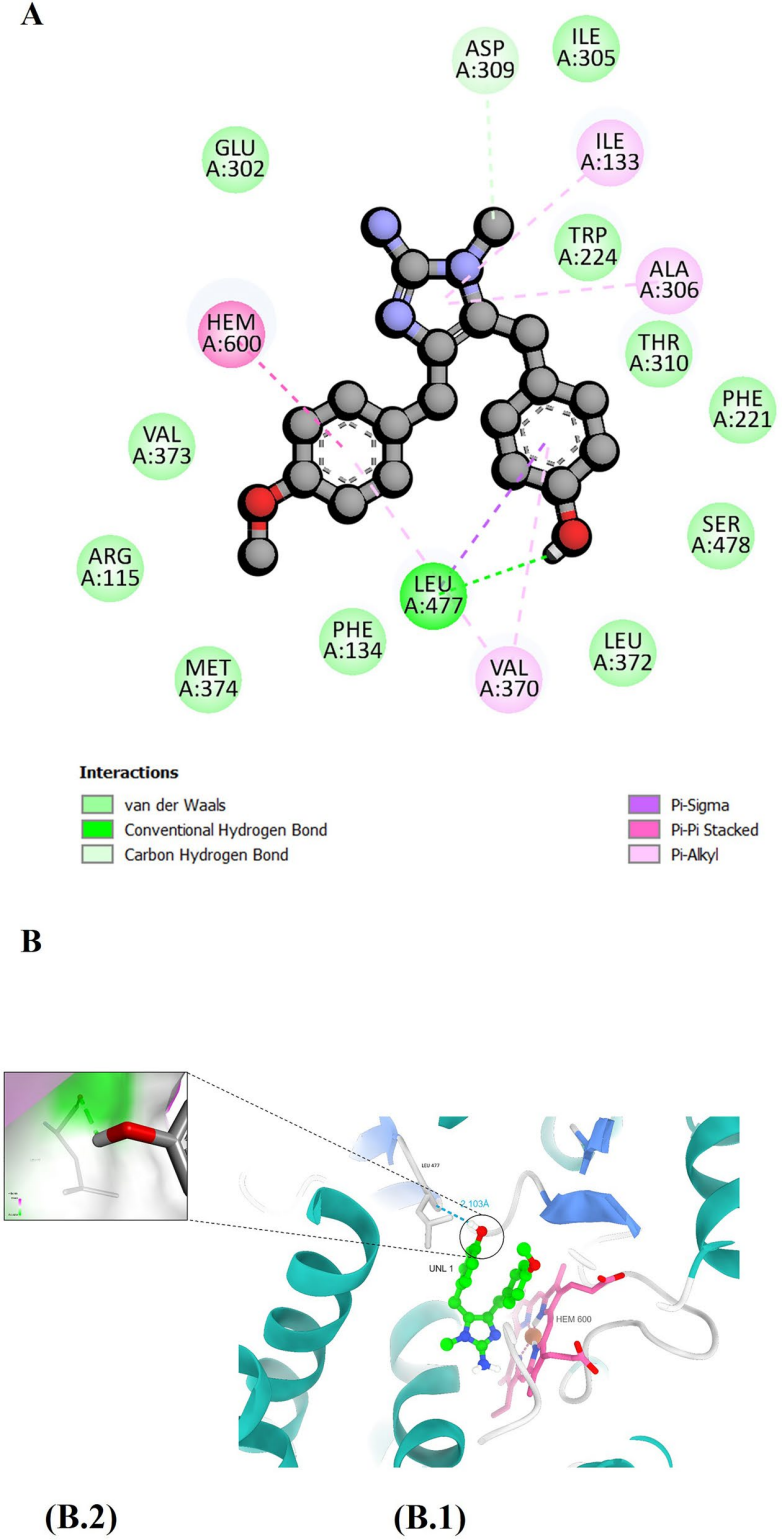
**A** A two-dimensional representation illustrating the interaction profile between CMNPD 11121 and the human aromatase enzyme. (**B**) illustrates hydrogen bond interactions within the binding site, with (B1) presenting an overview of the interactions and (B2) offering a close-up view of the hydrogen bond interaction along with a surface representation

The second hit, CMNPD11121, forms a single hydrogen bonding interaction with the Leu477 amino acid residue of the binding site (Fig. 7B). The following hydrophobic interactions take place between the ligand and the protein structures: Glu302, Val373, Arg115, Met374, Phe134, Leu372, Ser478, Phe221, Thr310, Trp224, and Ile305 (Fig. 7A).

**Fig. 8:**
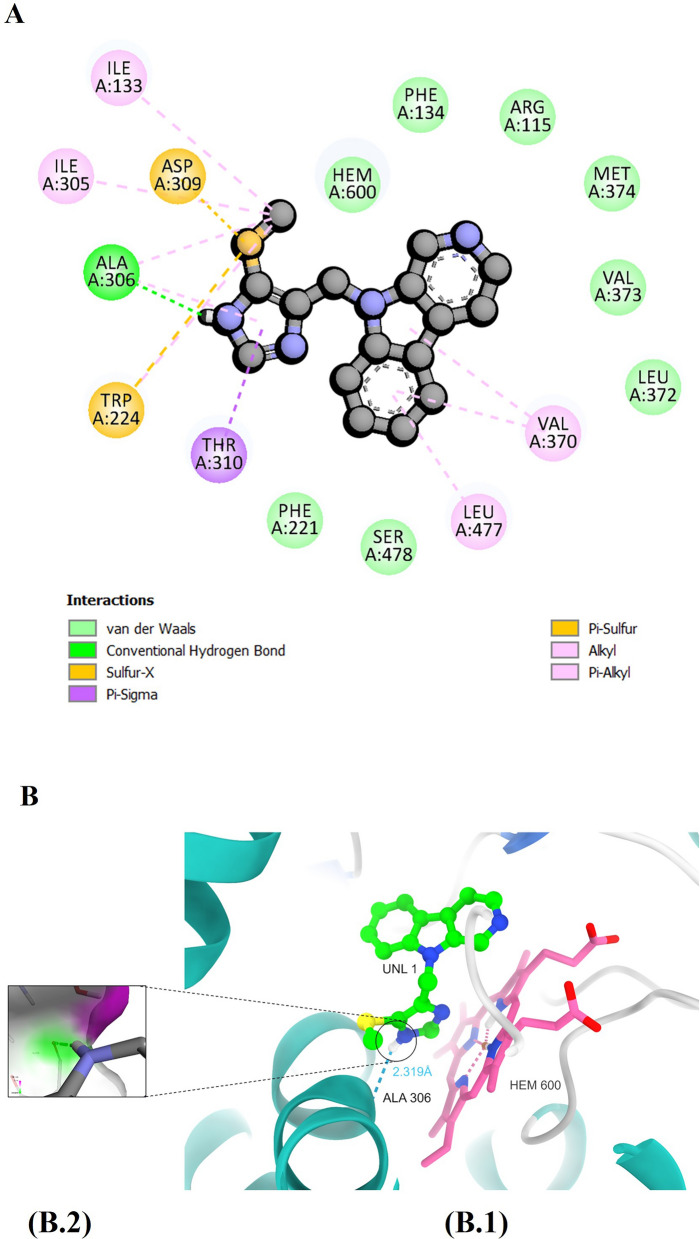
**A** Two-dimensional representation illustrating the interaction profile between CMNPD7905 and the human aromatase enzyme. **B** illustrates hydrogen bond interactions within the binding site, with (B1) presenting an overview of the interactions and (B2) providing a close-up view of the hydrogen bond interaction along with a surface representation

CMPND7905 forms a single hydrogen-bonding interaction with amino acid residue Ala306 (Fig. 8B). Favorable van der Waals non-bonded interactions include the heme moiety and amino acid residues Phe134, Arg115, Met374, Val373, Leu372, Ser478, and Phe221 (Fig. 8A).

**Fig. 9:**
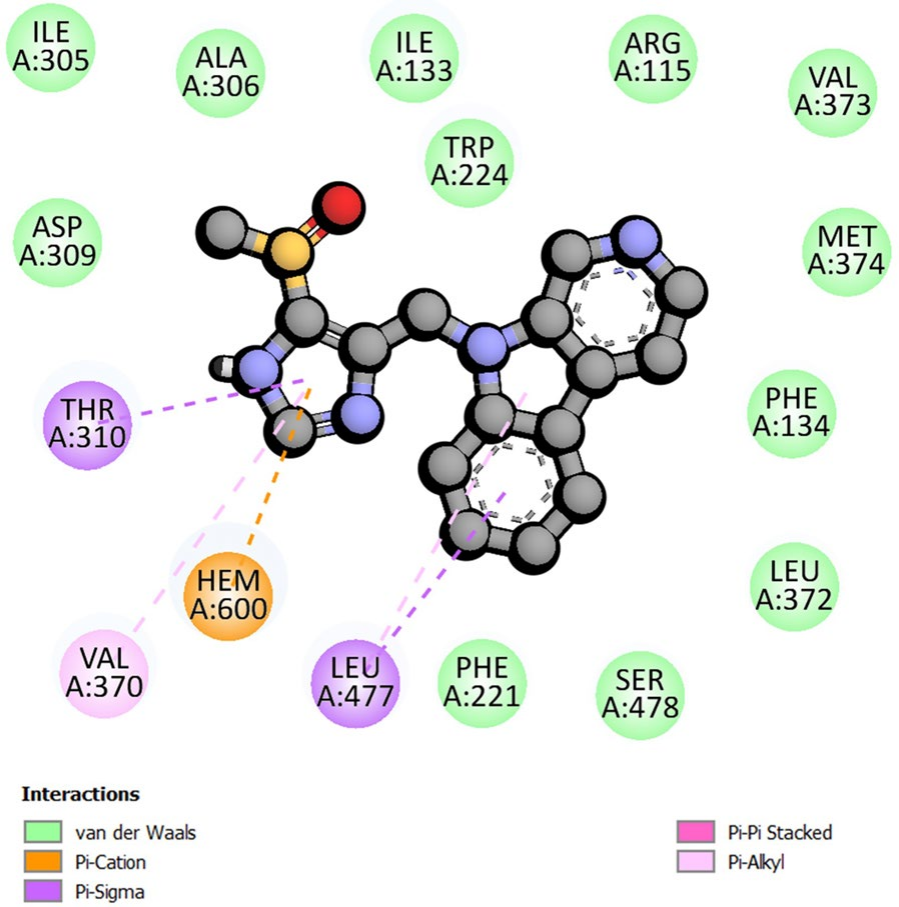
The figure depicts a two-dimensional representation illustrating the interaction profile between CMNPD7907 and the human aromatase enzyme

The protein–ligand interaction profile of CMNPD7907 shows no hydrogen bonding interactions within the protein–ligand complex (Fig. 9). The following amino acid residues participated in non-bonded van der Waal interaction between compound 7905 and CYP19A1: Val373, Arg115, Ile133, Ala306, Trp224, Ile305, Asp309, Phe221, Ser478, Leu372, Phe134, and Met374 (Fig. 9).

### Molecular dynamics simulation

**Fig. 10:**
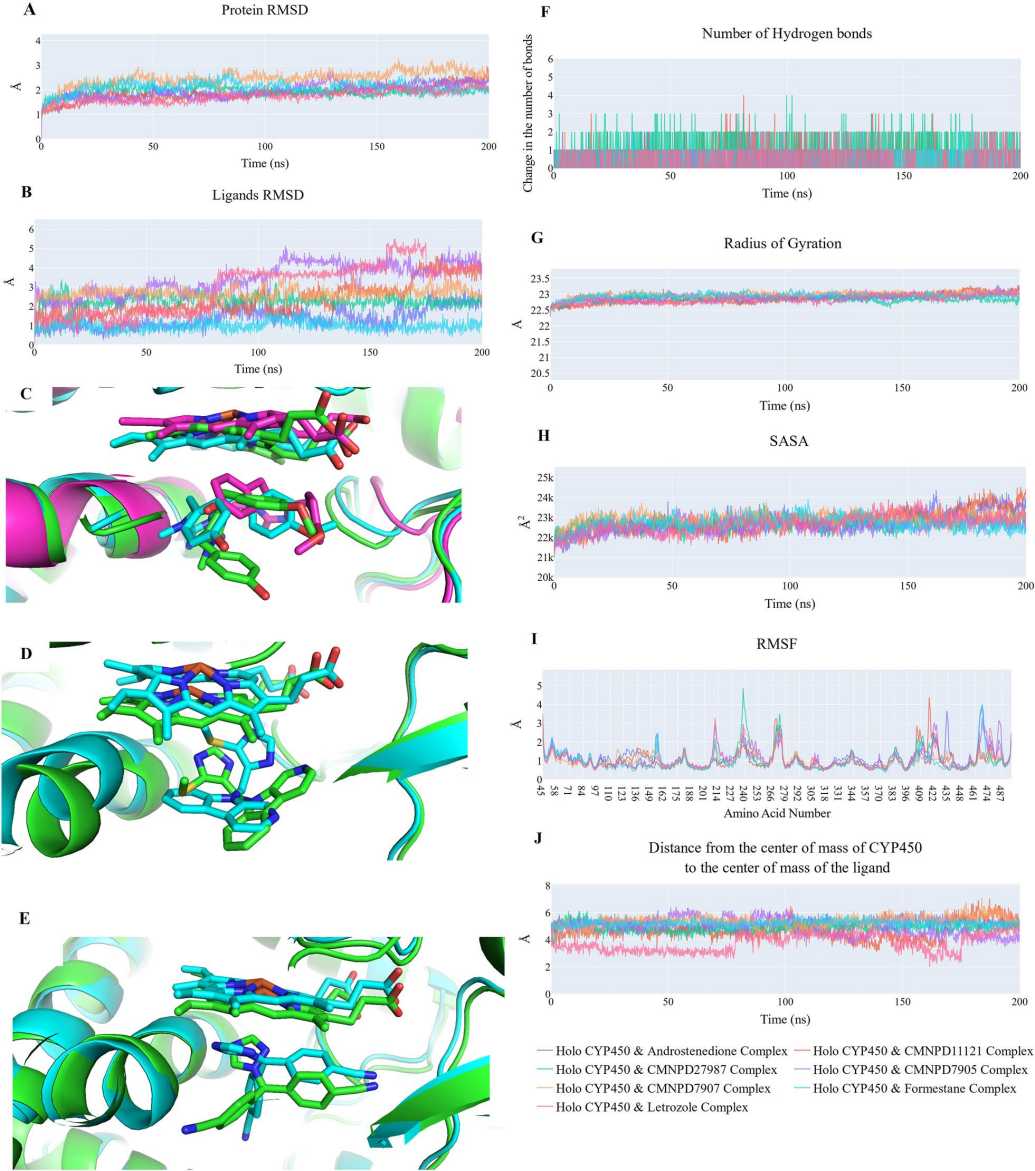
The figure shows (**A**) the protein RMSD values from each trajectory, **B** the ligands RMSD values, **C** a comparison between the structure of CMNPD11121 at 21 ns (green sticks), 155 ns (cyan sticks), and 192 ns (magenta sticks). The upper molecule is the HEME, **D** Shows a comparison between the structure of CMNPD7905 at 20 ns (green sticks) and 152 ns (cyan sticks), **E** Shows a comparison between the structure of Letrozole at 56 ns (green sticks) and 94 ns (cyan sticks), **F** change in the number of hydrogen bonds formed between CYP450 and each compound, **G** radius of gyration for the CYP450 in each system, **H** SASA for the CYP450 in each system, **I** RMSF for the CYP450 in each system and (**J**) distance from the center of mass of each ligand and CYP450 protein. In each figure, the CYP450 & Androstenedione system is the blue line, CYP450 & CMNPD11121 system is the red line, CYP450 & CMNPD27987 system is the green line, CYP450 & CMNPD7905 system is the magenta line, CYP450 & CMNPD7907 system is the orange line, CYP450 & Formestane system is the cyan line, and CYP450 & Letrozole system is the pink line

Throughout the production run, the compounds exhibited stable binding to the protein. In each system, protein structural integrity was stable, with RMSD values ranging from 1.7 Å to 2 Å from 25 ns, except for the CYP450 & CMNPD7907 complex, which had a slightly larger average of 2.5 Å (Fig. 10A). The ligand RMSD values (Fig. 10B) showed diverse patterns. Most compounds had stable RMSD values between 1 Å and 2.7 Å, with three exceptions. CMNPD11121 (red line) showed a stable trend for 130 ns, then increased to 2.8 Å (130–170 ns) and 4 Å in the last 30 ns. This change in RMSD value for CMNPD11121 indicated a conformational change, as shown in Fig. 10C, which compares its structure after 21 (green sticks), 155 (cyan sticks), and 192 (magenta sticks) ns. CMNPD11121 changed conformation and slightly deviated from its binding position. The second exception was CMNPD7905 (magenta line), which had two conformations in each simulation half. CMNPD7905 is shown in green at 20 ns and cyan at 152 ns in Fig. 10D. Between the two conformations, CMNPD7905 rotated around the protein while maintaining its binding location. Lastly, Letrozole (pink line) showed an average RMSD of 1.5 Å for 75 ns, then increased to 3.7 Å. Like CMNPD7905, a conformational change (rotation around the binding site) caused this rise. Figure 10E compares Letrozole's structure at 56 (green sticks) and 94 (cyan sticks) ns. The simulation showed that all compounds formed hydrogen bonds, including Androstenedione (blue line), Formestane (pink line), and Letrozole (cyan line). The fewest hydrogen bonds were in CMNPD7907 (orange line) and CMNPD7905 (magenta line). However, CMNPD11121 (red line) and CMNPD27987 (green line) formed two to three hydrogen bonds (Fig. 10F). Figure 10G and H reveal a consistent trend in the average radius of gyration (RoG) (22.8 Å) and solvent-accessible surface area (SASA) (22,500 Å2) for each system. All systems were stable, but molecules showed conformational changes. Additionally, C-alpha atom oscillations showed low values for most proteins in each system, with occasional spikes (> 2 Å) indicating loop fluctuations (Fig. 10I). In Fig. 10J, the distance between Androstenedione, CMNPD27987, CMNPD7907, and Formestane's center of mass and CYP450 remained stable at around 5 Å throughout the simulation. However, CMNPD11121, CMNPD7905, and Letrozole had slightly fluctuating values, which matched their RMSD values.

**Fig. 11:**
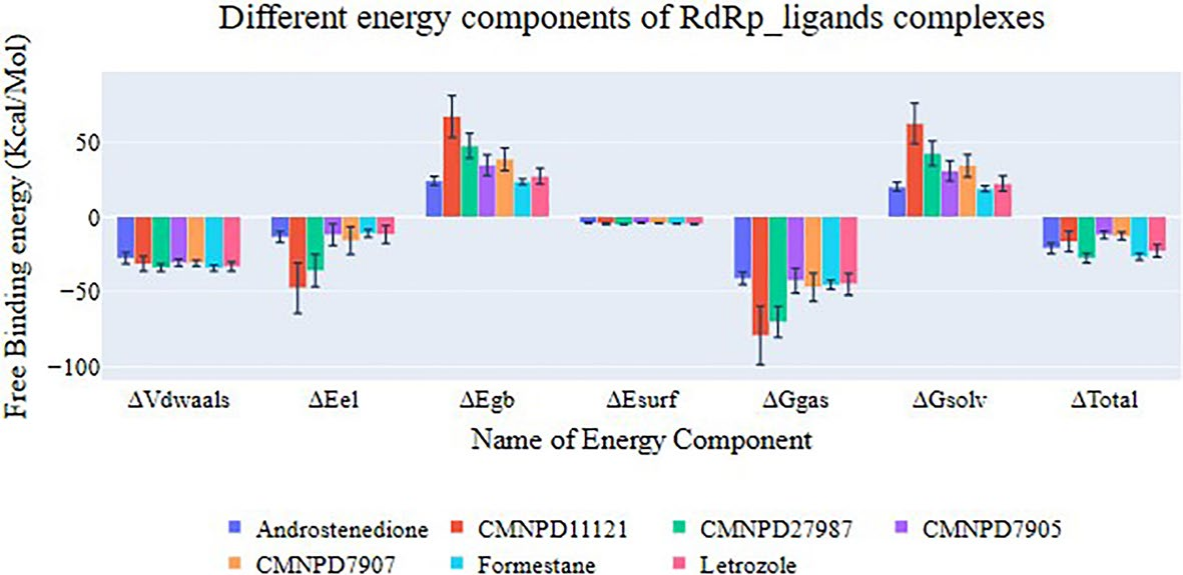
The figure shows the different energetic components of MM-GBSA and their values. Bars represent the standard deviations

The MM-GBSA-calculated binding free energy components has been shown in Fig. 11. CYP450's binding energies with Androstenedione (co-crystalized ligand), Formestane (commercially available drug), and Letrozole (commercially available drug) are −21.17, −26.75, and −22.82 kcal/mol, respectively. The two commercial drugs, especially Formestane, have a higher binding affinity than the co-crystal structure ligand. Only CMNPD27987 has a higher average total binding energy than the co-crystal ligand and currently used drugs, averaging −27.66 kcal/mol. The other three compounds, CMNPD11121, CMNPD7905, and CMNPD7907, have lower binding affinities than the co-crystal ligand, averaging −16.57, −12.14, and −12.84 kcal/mol, respectively. The van der Waals component averages −32 kcal/mol for most compounds, except Androstenedione, which averages −27.75. Electrostatic interactions show that the two compounds contribute more than the other ligands. CMNPD11121 has the highest average value of −47.66 kcal/mol, followed by CMNPD27987 at −36.03. Electrostatic contributions over −16 kcal/mol characterize the remaining molecules.

**Fig. 12:**
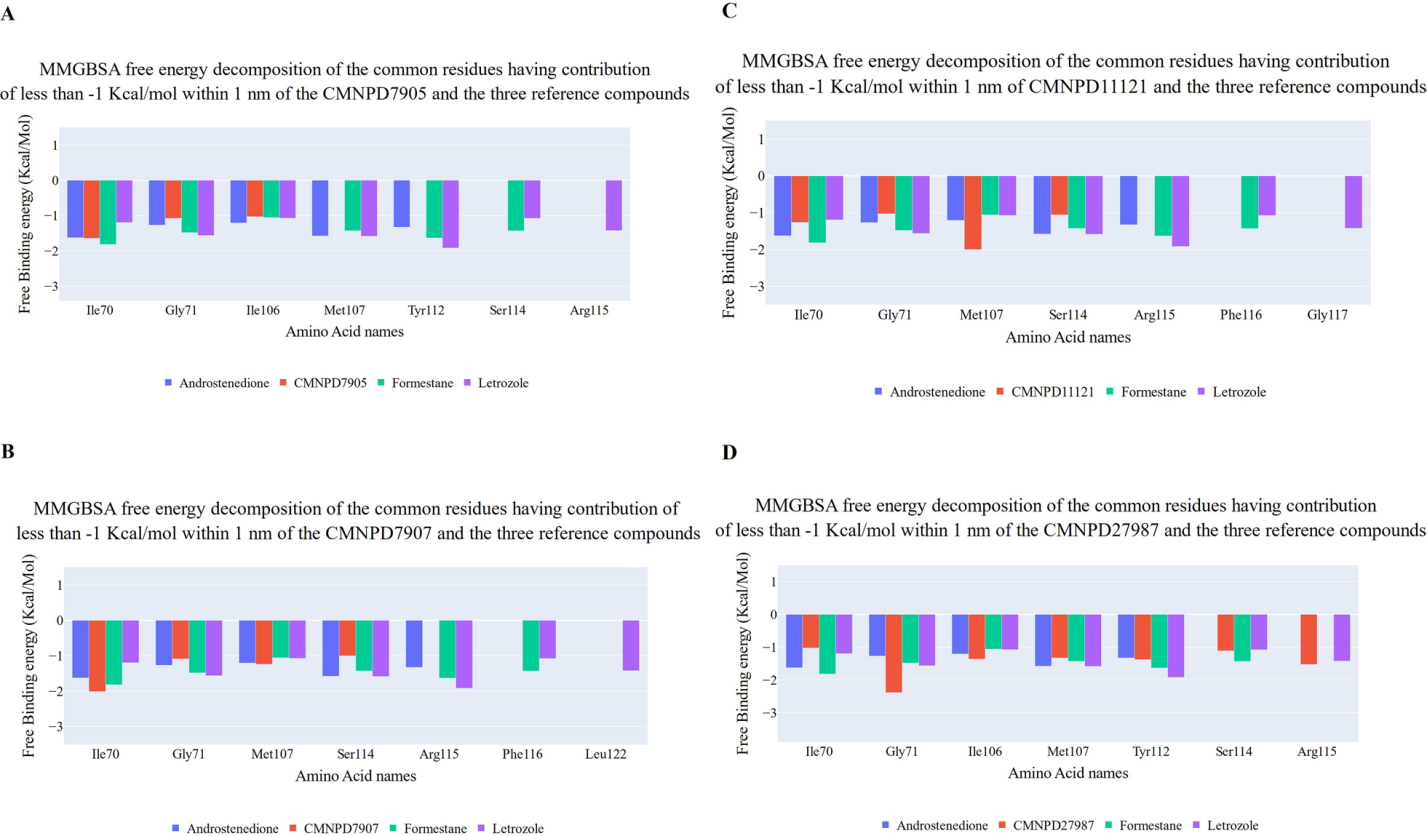
The figure shows the binding free energy decomposition of common amino acids between (**A**) CMNPD7905, (**B**) CMNPD7907, (**C**) CMPND11121, and (**D**) CMPND27987 and the three reference compounds (Androstenedione, Formestane, and Letrozole) that have a contribution of better than −1 kcal/mol. Amino acids with less than four bars mean that some compounds do not have a contribution of less than −1 kcal/mol

The decomposition of common amino acids within 1 nm of each compound compared to Androstenedione has been shown in Fig. 12A, B, C, and D, Formestane, and Letrozole. The reference compounds and CMNPD7905 (Fig. 12A) share six amino acids, three of which (Ile70 (−1.64 kcal/mol), Gly71 (−1.07 kcal/mol), and Ile106 (−1.02)) contribute −1 kcal/mol or less. The first amino acid outperforms Androstenedione and Letrozole, while the last two contribute less. Four amino acids (Ile70 (−2 kcal/mol), Gly71 (−1.08 kcal/mol), Met107 (−1.23 kcal/mol), and Ser114 (−1 kcal/mol)) contribute more than −1 kcal/mol to CMNPD7907 (Fig. 12B). CMNPD7907 binds better to Ile70 and Met107 than the reference compounds. The same four amino acids are in CMNPD11121 (Fig. 12C), but with different contributions. Met107 is the only amino acid that binds CMNPD11121 better than the reference compounds, averaging −1.99 kcal/mol. Finally, CMNPD27987 (Fig. 12D) shares seven amino acids with reference compounds: Ile70, Gly71, Ile106, Met107, Tyr112, Ser114, and Arg115 (−1.02–1.52 kcal/mol). This matches Fig. 11's best binding affinity.

**Fig. 13:**
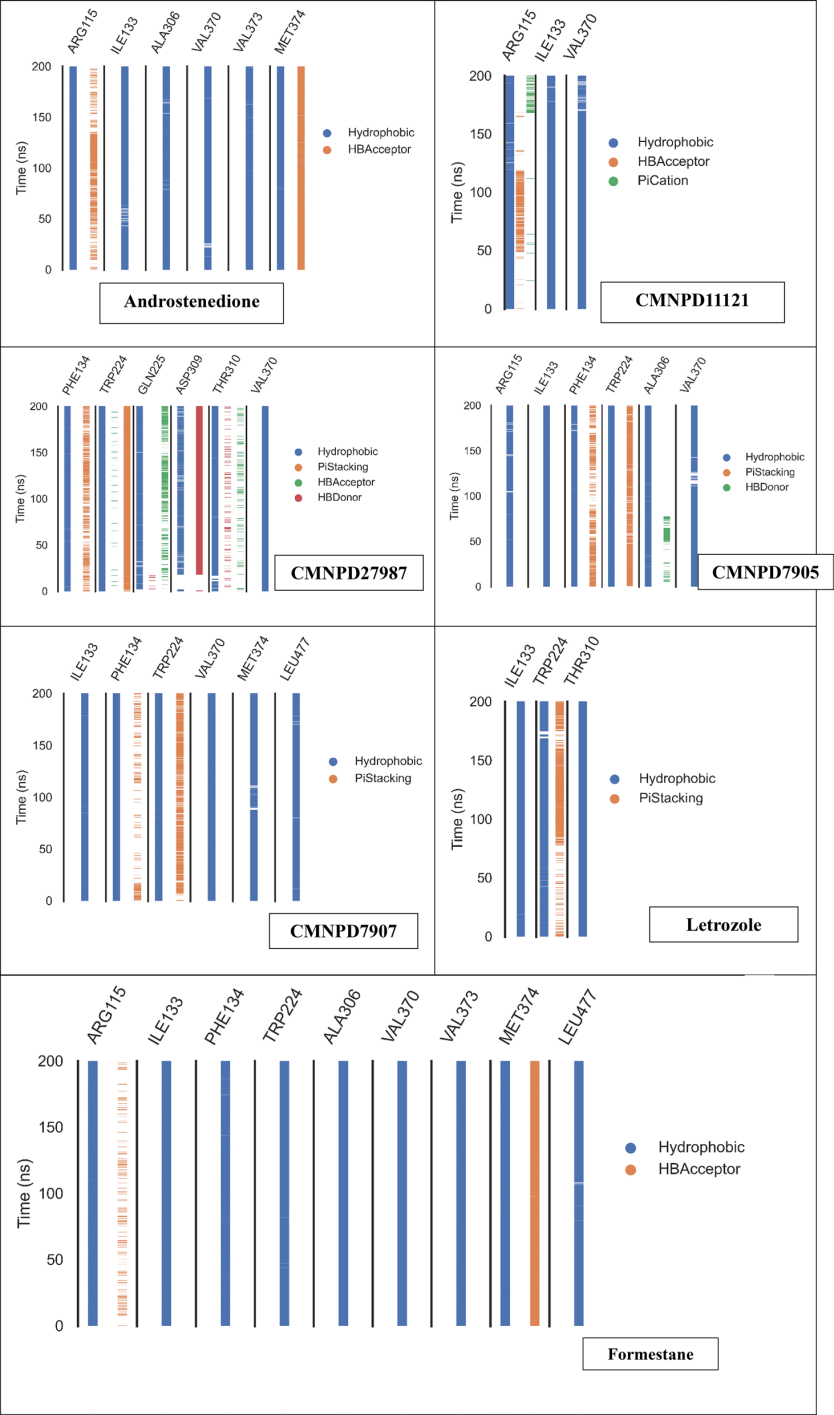
The figure shows the amino acids, the types of interactions with each of the seven ligands in this study, and their occurrence during the whole simulation time using the ProLIF Python library

As shown in Fig. 13, ProLIF library analysis revealed ligand-CYP450 binding interactions. The three reference compounds interacted consistently throughout the simulation, with occupancy rates of 92.3%. In Androstenedione, six amino acids had many hydrophobic interactions. Arg115 (97.3%), Ile133 (93.4%), Ala306 (92.3%), Val370 (95.3%), Val373 (96.1%), and Met374 (98.1%). Met374 also formed 94.1% hydrogen bonds. Formestane also had nine hydrophobic interactions with frequencies of at least 95.3%. Arg115 (97.9%), Ile133 (99.8%), Phe134 (95.3%), Trp224 (95.8%), Ala306 (99.6%), Val370 (99.4%), Val373 (99.9%), Met374 (96.7%), and Leu477 (95.3%) Like the co-crystalized ligand, Met374 formed hydrogen bonds at 96.6%. Letrozole had hydrophobic interactions with Ile133 (98.9%), Trp224 (93%), and Thr310 (99.2%).

The remaining four compounds involved hydrophobic interactions with CMNPD11121, Androstenedione, and Formestane via three amino acids: Arg115 (93.8%), Ile133 (97.2%), and Val370 (93.8%). CMNPD7905 also interacted hydrophobically with six amino acids: Arg115 (90.2%), Ile133 (99.6%), Phe134 (94.4%), Trp224 (99%), Ala306 (95.3%), and Val370 (91.9%) Those interacting with Formestane share these amino acids. CMNPD7907 hydrophobically interacted with six amino acids: Ile133 (96.3%), Phe134 (99.3%), Trp224 (97.6%), Val370 (99.8%), Met374 (95.3%), and Leu477 (94.2%). Often compared to Formestane-interacting amino acids. Finally, CMNPD27987 interacted in many ways. The amino acids Phe134 (94.2%), Trp224 (100%), Gln225 (91.1%), Thr310 (94.6%), and Val370 (98.4%) formed hydrophobic interactions. Pi-stacking occurred 93.9% of the time for Trp224. A hydrogen bond occurred 90.8% of the time with Asp309.

## Discussion

The progression of carcinogenesis in hormone receptor-positive breast cancer patients can depend on the peripheral conversion of androgen precursors to estrogen through the human aromatase enzyme [4]. Considering the predominant hormone dependence of most breast cancer tumors [49], CYP19A1 offers an important avenue for the investigation of new lead candidates capable of inhibiting estrogen biosynthesis [50]. We used computational methods to find marine natural products that bind to the human aromatase enzyme's active site [51–53].

The best ligand-based pharmacophore model scored 0.94. This model had the highest pharmacophore-fit score with Compound 6, Kang et al.'s most potent compound. [24]. An in-house benchmarking database of 40 actives from the literature and 815 decoys generated by the DecoyFinder program was used in each step of our pharmacophore modeling protocol. [54–56]. The model produced 3 hits, all of which were true positives, but its low sensitivity (7.5% of total actives) offset its high specificity, making it unsuitable for virtual screening. Due to the lack of enzyme binding site structure in ligand-based approaches [57], the model failed in revealing crucial amino acid residues that complement the pharmacophoric features of the ligand. To overcome the absence of crystallographic, experimental structures for protein–ligand complexes, researchers often resort to docking studies, utilizing either homology models or existing crystal structures [58–60]. Molecular docking helps identify bioactive poses for candidate ligands by exploring conformational space.

We used AutoDockVina to study the interaction between the azaheterocyclic ring and the iron porphyrin's heme atom in the enzyme's active site to design a structure-based pharmacophore mode for Compound 6 and letrozole. As a widely studied non-steroidal drug, letrozole was used as a reference [27, 61–65]. UV-absorption spectrum data suggests that letrozole's correct binding pose at aromatase's binding site must accommodate the metal coordination bond between its proximal nitrogen and the iron porphyrin's heme atom. Our docking study generated the correct pose, but AutoDockVina did not rank it highest. Our findings contradict several published studies on letrozole and other azaheterocylic ring-containing compounds' binding to human aromatase. [9, 24, 64, 66, 67]; however, they are in line with a recent computational study that revealed Vina, among other commonly used docking programs, is not accurate in ranking the output poses of ligands docked to metalloproteins [68]. We found similar docking results to Galeazzi and Massaccessi's Autodock 4.0 study. The correct pose of the azaheterocyclic ring facing the heme atom was confirmed by a molecular dynamic study. [27]. Some studies introduced a distance constraint during docking to account for the metal coordination bond between the heme iron and the proximal nitrogen of the ligand [69–71]. Not doing so allowed us to fully explore the conformational space for an energetically favorable orientation. Our group used molecular docking through AutodockVina to generate poses for Compound 6 and letrozole. Using clustering analysis, RMSD value for azaheterocyclic ring of representatives from each cluster and for the bioactive pose of letrozole proposed in the literature, and the distance between the Fe atom and the representative confirmation from each cluster, we were able to manually select potential bioactive poses. Ourt structural based model established based on the aforementioned criteria has shown interactions with amino acid residues like Met374, Phe134, Val373, Thr310, and Leu477. [9, 11, 61, 63–65, 72, 73]. The model, however, showed low specificity producing 5 false positives (AUC 0.72 and EF 16.7) despite its high sensitivity. The combination of structure- and ligand-based methods in drug discovery is a growing approach in CADD [74]. Employing an integrated strategy can enhance the strengths and mitigate the limitations of each method [75]. Smith et al. devised a sequential pipeline that involves refining hits obtained from multiple ligand- and structure-based pharmacophores by applying druglikeness and ADMET filters [59]. Malgorzara et al. utilized a similar approach to retrieve DNA topoisomerase I inhibitors [76]. To save computational power and virtual screening time, we combined ligand-based and structural-based pharmacophore models instead of sequential approaches. The model used structural knowledge of the protein's binding pocket and the common pharmacophoric features of promising ligands. A similar approach was employed in two previous studies: in the firt, a ligand- and structure-based pharmacophore model were merged to improve virtual screening for cancer. Osaka thyroid (COT) kinase inhibitors using LigandScout [77]. The second study included a similar regimen to detect CDK9/Cyclin T1 kinase inhibitors [78]. Both studies lack benchmarking validity to assess the model's bioactive lead candidate identification efficiency, unlike ours. Merging the pharmacophoric features of both models improved sensitivity and specificity to 0.77 and 21.4, respectively. The model produced 22 true positives, representing 55% of total actives.

The aromatase-androstenedione complex showed hydrogen bonding with Met374 and Arg115. The ligand additionally interacts via van der Waals forces with residues Phe221, Leu477, Phe134, Leu372, Val373, Val370, Ile133, Thr310, Ile305, and Asp309. This confirms previous findings. [79, 80]. According to Ghosh et al., the steroidal substrate's mechanism involves catalytic residues Thr310, Ala306, and Asp309. Thr310 and Ala306 attack H2β carbon, while protonated Asp309 promotes 2,3-enolization by electrophilic attack on 3-keto oxygen. Their research showed that steroidal inhibitors like exemestane could stop aromatization by reducing Thr310 mobility through favorable interactions. [80]. ASD had a stable protein and ligand RMSD, radius of gyration, SASA, and distance between its center of mass and CYP19A1 during the 200 ns molecular dynamics simulation. According to ProLIF library data, ASD formed stable hydrogen bonds with Arg115 and Met374. Given its overall favorable interactions, it had one of the highest MM-GBSA free binding energies at −21.17 kcal/mol. It also showed six hydrophobic interactions with amino acids Arg115 (97.3%), Ile133 (93.4%), Ala306 (92.3%), Val370 (95.3%), Val373 (96.1%), and Met374 (98.1%), three of which were confirmed by molecular docking.

We found that formestane, another steroidal inhibitor with a C6 hydroxy group, hydrogen bonds with amino acid residues Arg115, Met374, and Asp309. Suvannang et al. suggested that formestane can form hydrogen bonds with Met374's backbone amine and Asp309's carboxylic acid, resulting in tighter and stronger van der Waals contacts with surrounding amino acids and a high aromatase binding affinity. [11]. Formestane may disrupt the proton relay network that aromatizes androgen precursors by binding to Asp309. Molecular dynamics simulation showed low protein and ligand RMSD values, confirming formestane's stability in protein complexes. According to ProLif library data, Formestane formed at least one hydrogen bond with Met374 in the simulation, supporting our molecular docking findings. A stable radius of gyration, SASA, and distance between its center of mass and the aromatase protein were also observed. At −26.75 kcal/mol, formestane had the second-highest MM-GBSA free binding energy of all tested hits, indicating stable binding. Our molecular dynamics simulation confirmed four of formestane's nine hydrophobic interactions with the aromatase enzyme's backbone, which suggests its stability. ProLIF library analysis suggests at least 95.3% of these interactions occur.

Nonsteroidal aromatase inhibitors reversibly coordinate the heme moiety's iron atom through the heterocyclic ring's distal nitrogen. Letrozole, the reference non-steroidal AI used in this study, formed hydrogen bonds with residues Met374 and Arg115 through one of its two benzonitrile groups and a third bond with Ser478 through the other moiety, suggesting a role in the aromatization reaction's first and second hydroxylation steps. [81]. Also observed were van der Waals contacts with amino acids His480, Val369, Ser478, Leu477, Phe134, Val373, Trp224, Phe221, Asp309, Thr310, HOH630, and Val313. Letrozole's triazole ring stacks Pi-Pi with the porphyrin ring. Previous research supports these findings [71, 82, 83]. Hydrogen bonding with a benzonitrile group or another strategically positioned moiety relative to the azaheterocyclic ring is recognized as crucial for achieving potent aromatase inhibitory effects in nonsteroidal aromatase inhibitors [84, 85]. Protein RMSD in molecular dynamics simulations showed letrozole-stabilized CYP19A1. In the first 75 ns, letrozole's ligand RMSD was stable but then fluctuated to 3.7 Å. These findings suggest that letrozole may have changed conformation to correct its active site binding pose. Despite rotation, the ligand maintained its azaheterocyclic ring toward the heme moiety. The simulation showed that letrozole formed at least one hydrogen bond, supporting the molecular docking results. Due to fluctuations, letrozole maintained a stable radius of gyration and SASA but not a consistent distance between its center of mass and CYP19A1. Letrozole's MM-GBSA free binding energy was −22.82 kcal/mol, the third highest among all compounds and higher than the co-crystalized ligand, indicating robust binding. Letrozole's stability was confirmed by ProLIF library analysis, which showed hydrophobic interactions with three amino acids: Ile133 (98.9%), Trp224 (93%), and Thr310 (99.2%). All molecular docking results agree.

Among the most stable active hits, CMPND 27987, an imidazole ring faces the iron atom of the heme moiety at 5.4 Å. One of the two phenol groups forms hydrogen bonding interactions with catalytic residues Ser478, HOH605, and Met374, Arg115 in the catalytic cleft, similar to the two benzonitrile moieties of letrozole. Besides van der Waal interactions with amino acid residues Leu372, Arg115, Phe134, Val313, His480, Val369, Phe221, Asp309, Leu477, and Trp224, CMPND 27987 forms hydrophobic contacts with the iron porphyrin. These positive interactions may explain CMPND 27987's high aromatase enzyme binding affinity. Protein and ligand RMSD data from the molecular dynamic simulation showed that CYP19A1 and CMPND 27987 remained stable. The ligand formed 2–3 hydrogen bonds with the protein's backbone the most. Despite molecular docking results, ProLIF library analysis shows CMPND 27987 forming hydrogen bonds with amino acids Trp224, Gln225, Asp309, and Thr310, which is consistent with literature data for other aromatase inhibitors. [11, 86]. It kept its solvent-accessibility surface area and gyration radius stable. It also had a stable center of mass distance from CYP19A1. CMPND27987 had the highest MM-GBSA free binding energy of −27.75 kcal/mol, higher than the co-crystallized ligand, indicating strong binding affinity. It shares seven amino acids with the three reference compounds used in this study (Ile70, Gly71, Ile106, Met107, Tyr112, and Arg115). The second-highest average value of −36.03 kcal/mol for electrostatic interactions shows that CMNPD27987 contributes more than most other ligands. Molecular docking studies confirmed that two of the five amino acids—Phe134 (94.2%) and Trp224 (100%), Gln225 (91.1%), Thr310 (94.6%), and Val370 (98.4%)—formed hydrophobic interactions with CMPND 27987. Trp224 also engaged in Pi-stacking interactions with a 93.9% rate, which was not supported by molecular docking but agreed with Galeazzi and Massaccesi's letrozole results. [27].

The phenol group of CMPND 11121 forms a single hydrogen bonding interaction with Leu477, which has been suggested to induce therapeutic potential [87]. It forms a Pi-Pi stacked interaction with the heme moiety and van der Waal interactions with residues Glu302, Val373, Arg115, Met374, Phe134, Leu372, Ser478, Phe221, Thr310, Trp224, and Ile305. CMPND 11121's lower binding affinity than CMPND 27987 may be due to its inability to hydrogen bond with Met374. Molecular dynamics simulations showed that CYP19A1 and CMPND 11121 remained stable throughout the study, as shown by consistent protein RMSD data. In the simulation, CMPND 11121 ligand RMSD was stable for 130 ns but increased to 2.8 Å between 130 and 170 ns and 4 Å in the final 30 ns. The final conformation shows the azaheterocyclic ring 0.7 nm from the center of the heme molecule, suggesting CMPND 11121 may not coordinate the iron atom. Like CMPND 27987, CMPND 11121 had 2–3 hydrogen bonds with the protein's backbone. ProLIF library analysis showed one hydrogen bond with Arg114, matching docking results. The ligand maintained a stable radius of gyration and SASA, but its center of mass and that of CYP19A1 fluctuated slightly, which could be explained by the conformational change. CMPND27987's MM-GBSA free binding energy was −16.57 kcal/mol, which was lower than previous hits, suggesting that the conformational change may prevent the ligand from coordinating into heme's iron atom. Like the three reference compounds used in this study, CMPND 11121 interacts with four key amino acids: Ile70, Gly71, Met107, and Ser114. Electrostatic interaction analysis showed that CMPND27987 contributed the most, averaging −47.66 kcal/mol. The hydrophobic interactions of three amino acids—Arg115 (93.8%), Ile133 (97.2%), and Val370 (93.8%)—with CMPND 11121 were confirmed by molecular docking studies and also observed in androstenedione and formestane.

The third hit, CMPND 7905, was found to form one hydrogen bond with the amino acid residue Ala306, another catalytic cleft key residue involved in the hydrophobic stabilization of the ligand–protein interaction [88]. It also forms hydrophobic interactions with important residues like Thr310, Ala306, Ile133, Ile305, Trp224, Val370, Ala306, Val370, and Leu477 and Van der Waals contacts with amino acids Phe134, Arg115, Met374, Val373, Leu372, Ser478, and Phe221. CMPND 7905 forms a sulfur-X bond with residue Asp309 and a Pi-Sulfur interaction with Trp224's ring. Molecular dynamics simulations showed that CYP19A1 and CMPND 7905 maintained stability throughout the study, as shown by consistent protein RMSD data. CMPND 7905's ligand RMSD shows a rotational change relative to the active site while maintaining binding. Less than one hydrogen bond was formed by CMPND 7905 with the protein backbone. In the first 100 ns of the study, ProLIF library analysis found a hydrogen bond with Val370, supporting docking results. CMPND 7905 maintained a stable radius of gyration and SASA, but its center of mass distance from CYP19A1 fluctuated due to the conformational change. Due to its low hydrogen bond count, CMPND 7905 had the lowest MM-GBSA free binding energy at −12.14 kcal/mol. Compared to the three reference compounds, CMPND 7905 shared six amino acids (Ile70, Gly71, Ile106, Met107, Ser114, and Arg115) within 1 nm. The first three were common to all reference compounds. Arg115 (90.2%), Ile133 (99.6%), Phe134 (94.4%), Trp224 (99%), Ala306 (95.3%), and Val370 (91.9%) had hydrophobic interactions.

The final hit, CMPND 7907, did not form hydrogen or carbon-hydrogen bonds with human aromatase-binding site amino acid residues. It bonded hydrophobically to Thr310, Leu477, Hem600, and Val370. Several Van der Waals interactions stabilized the ligand in the active site, including amino acids Val373, Arg115, Ile133, Ala306, Trp224, Ile305, Asp309, Phe221, Ser478, Leu372, Phe134, and Met374. The ligand and heme moiety interacted electrostatically. Molecular dynamics simulations showed that CYP19A1 and CMPND 7907 had stable protein and ligand RMSD values throughout the study. Docking predictions were met when the compound formed less than one hydrogen bond with the protein's backbone, like CMPND 7905. The compound's radius of gyration SASA and distance from CYP19A1's center of mass were stable. CMPND 7907 had the second-lowest MM-GBSA free binding energy at −12.84 kcal/mol, possibly due to limited hydrogen bonding. Comparing amino acids within 1 nm of each compound to three reference compounds revealed four common amino acids for CMPND 7907 (Ile70, Gly71, Ile106, Met107, and Ser114), with the first three shared by all references. Ile133 (96.3%), Phe134 (99.3%), Trp224 (97.6%), Val370 (99.8%), Met374 (95.3%), and Leu477 (94.2%) had hydrophobic interactions with formestane, which molecular docking studies confirmed.

## Conclusions

Inhibition of the human aromatase enzyme is a promising therapy to combat estrogen-receptor positive breast cancer. In this study, we used a merged pharmacophore model derived from the 3D structure of 3EQM and a promising series of ligands containing azaheterocyclic rings to screen a marine natural product database for potential inhibitors. Among 1,385 identified compounds, four candidates emerged, and through molecular docking and dynamics studies, CMPND 27987 proved to be the most stable. The compound had the highest docking score among the selected candidates and the highest MM-GBSA free binding energy across the board. It was able to form the highest number of hydrogen bonds (at least 3) with the protein’s amino acid backbone, in addition to several favorable hydrophobic interactions. Molecular dynamics simulations showed stable interactions between CMPND 27987 and CYP19A1, supported by several parameters. This study suggests that further research, including pharmaceutical development and preclinical studies, could advance CMPND 27987 toward clinical trials as a potential therapy for the human aromatase enzyme.

## Supplementary Information

Additional file 1.

## Data Availability

The data supporting the findings of this study are provided within the main text and its supplementary materials. The benchmarking dataset and any raw data in various formats are available from the corresponding author upon reasonable request.

## Abbreviations

ER: Estrogen receptor
CYP19A1: Human aromatase enzyme
AI: Aromatase inhibitor
IC50: Half maximal inhibitory concentration
RMSD: Root mean square deviation
