# Supplementary material for: Pharmacophore-based virtual screening, molecular docking, and molecular dynamics investigation for the identification of novel, marine aromatase inhibitors
Source: BMC Chem. 2024 Nov 26;18(1):235. doi: 10.1186/s13065-024-01350-9 (PMC11590544; doi:10.1186/s13065-024-01350-9)
Supplement: Supplementary file 1 — Additional file 1. [file 13065_2024_1350_MOESM1_ESM.docx]

**Supplementary Material**

**Pharmacophore-Based Virtual Screening, Molecular Docking, and Molecular Dynamics Investigation for the Identification of Novel, Marine Aromatase Inhibitors**

**Cluster analysis for Compound 6 and Letrozole**

For this study, the selection of bioactive pose candidates was based on a three-step scheme in which molecular docking generated poses for both Compound 6 and letrozole were subjected to a cluster analysis. Secondly, the root mean square deviation (RMSD) value was calculated for the azaheterocyclic ring of representatives from each cluster, as well as for the bioactive pose of letrozole suggested in the literature. Finally, the distance between the Fe atom in the heme moiety and the azaheterocyclic ring of the representative confirmation from each cluster was determined. The cluster representative showing optimal binding energy, lowest RMSD, and shortest distance to heme would be selected for structure-based pharmacophore modeling.

Each docking iteration generated multiple poses for both letrozole and Compound 6, resulting in a diverse set of conformations for each compound. The BioPandas library in Python was utilized to calculate RMSD between every pair of poses generated for each compound [1]. A distance matrix was constructed for hierarchical clustering, which grouped similar conformations based on their structural similarity using complete linkage with a cutoff of 2 Å.

Lastly, taking into account the mean binding energy of each cluster and the number of conformers associated with it, the representative pose, which had the lowest distance to the Fe atom in the heme porphyrin, as well as the lowest RMSD was selected for constructing the structural-based pharmacophore model using the default parameters of LigandScout.

Similar to the structure-based pharmacophore model, the selection of the bioactive pose candidate was made manually utilizing hierarchical cluster analysis of poses produced after 100 iterations, RMSD calculation between the azaheterocylic ring of cluster representatives and that of letrozole, and distance between the azaheterocyclic ring center and the Fe atom of the heme group.

For the docking-assisted structural-based pharmacophore model, both Compound 6 and letrozole underwent 100 instances of molecular docking to the active site of the human aromatase. To select the potential bioactive pose on which the model will be based, the pool of resulting poses was subjected to clustering analysis. In the case of letrozole, the distance from the Fe atom in the heme moiety and the triazolic ring was determined for representatives of each cluster.

For letrozole, clustering analysis showed the presence of five potential poses. The biggest two clusters exhibited notable spatial separation from the Fe atom of the iron porphyrin of the active site. The first cluster, which had the highest number of conformers (169) and the second-highest binding affinity (-5.94 Kcal/mol), showed the triazole moiety pointing away from the Fe atom of the heme moiety with a distance of 7.7 Å between the pair. Similarly, the second cluster, with the highest binding affinity (-6.2 Kcal/mol) and the second highest number of conformers (122 conformers), had its azaheterocyclic ring at an 8.0 Å distance from the metal center. Only the third cluster (75 conformers and binding affinity of -5.69 Kcal/mol) had its triazolic ring directed to the heme moiety, which was in line with the bathochromic shift of the Soret band observed in UV spectrum data. This pose was also in agreement with the molecular dynamics study of Galeazzi and Massaccesi. Accordingly, it was selected as a reference pose to aid in the selection of Compound 6’s probable bioactive pose on which the structural-based model will be based (Fig. S1).


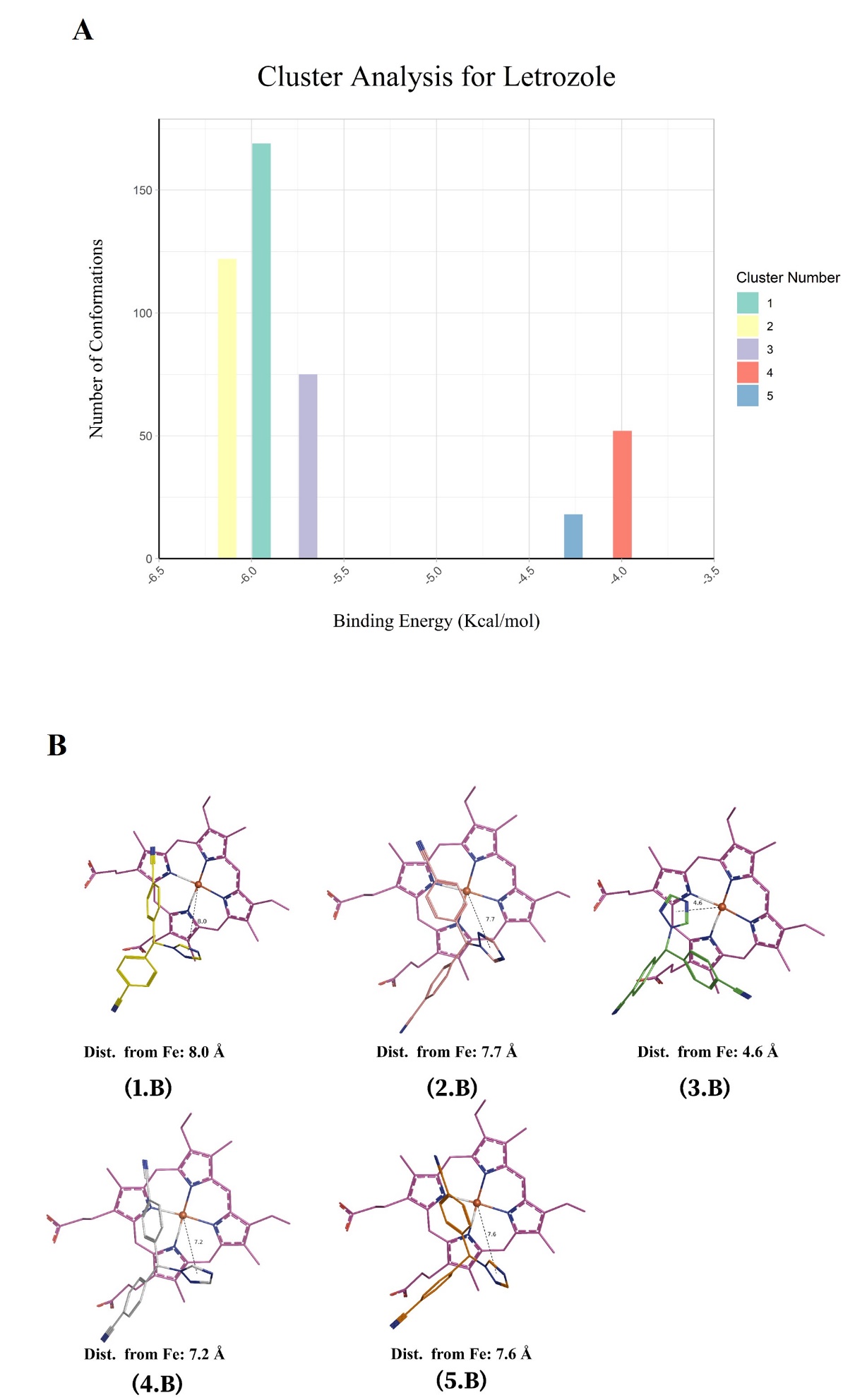


Fig. S1. The figure shows (A) the results of cluster analysis for letrozole, (B) the RMSD value of the azaheterocylic ring of each cluster representative compared to that of letrozole, and (C) distances between the azaheterocyclic ring and the Fe of the heme group.

Clustering analysis for Compound 6 resulted in five poses, in which Cluster 2 had the highest number of conformers at 217 and the best mean binding energy (-5.94 Kcal/mol); however, its azaheterocyclic ring showed an RMSD of 6.98 when compared to that of letrozole and a distance of 8.0 Å from the metal center of the binding site. Despite having a relatively low number of conformers and mean binding energy, Clusters 3 (100 and -4.073 Kcal/mol) and 4 (109 and -4.5 Kcal/mol) displayed favorable RMSD values of 1.79 and 1.87, along with distances of 4.6 Å and 3.9 Å, respectively, which positioned them as promising candidates for constructing the structural-based pharmacophore model (Fig. S2). Upon viewing Cluster 3 and 4 in their respective complexes with the human aromatase enzyme in the Structure-Based view of LigandScout, only Cluster 3 showed an iron-binding moiety directed toward the Fe atom of the heme center, and therefore, it was selected for constructing the structural-based pharmacophore model (Fig. S2 3 and 4C).


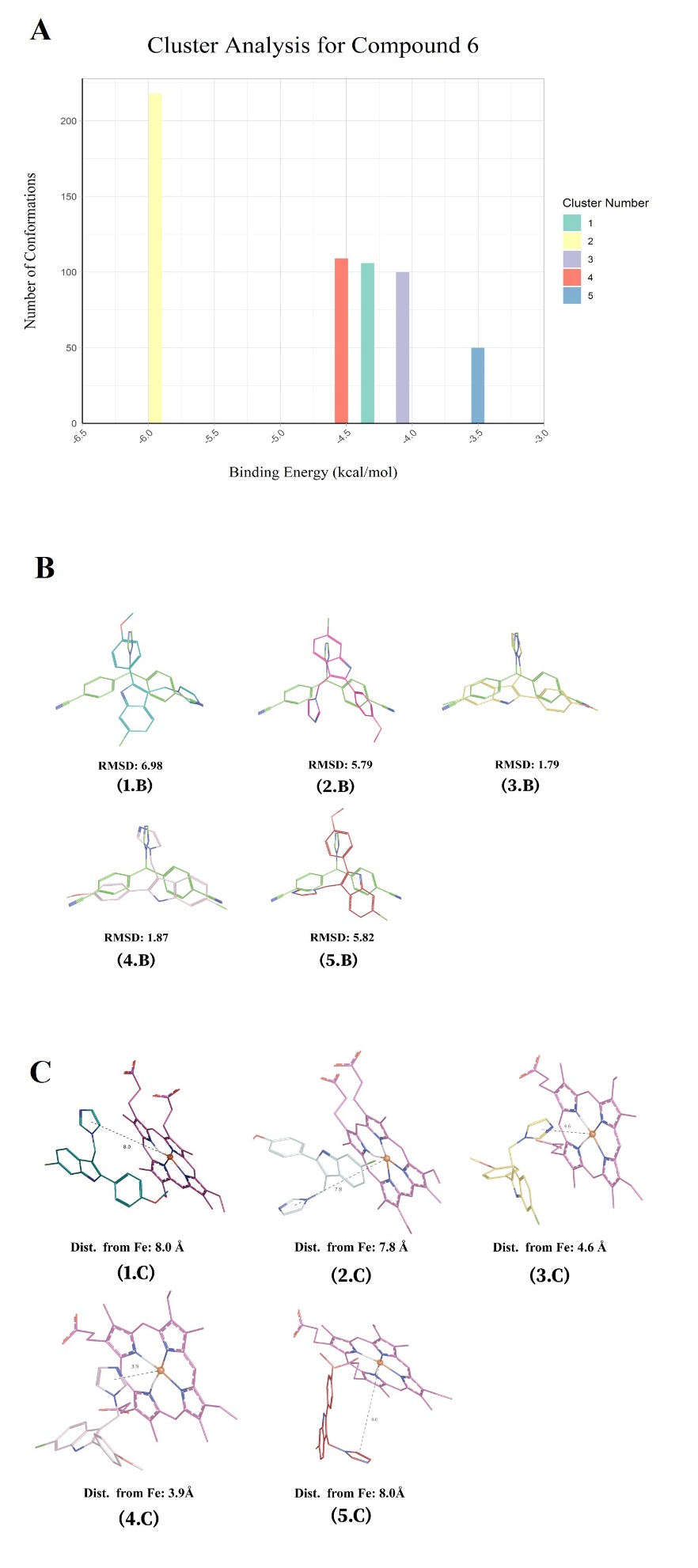


Fig. S2. The figure (A) the results of cluster analysis for Compound 6, (B) the RMSD value of the azaheterocylic ring of each cluster representative compared to that of letrozole, and (C) distances between the azaheterocyclic ring and the Fe of the heme group.

**Cluster analysis for molecular docking results**

For the first compound, CMNPD27987, a total of 7 clusters were identified. Two clusters had structural similarities to the binding pose of letrozole used as a reference. Cluster 4 had a total of 75 conformations and an average binding energy of -7.37 Kcal/mol. Additionally, its azaheterocyclic ring had an RMSD of 1.99 Å when compared to that of the reference, while the distance from the Fe atom at the metal binding center was recorded at 5.4 Å. Cluster 5 scored better with a total of 100 conformers, -7.47 Kcal/mol mean binding energy, RMSD value of 1.99 Å, and a distance of 5.4 Å. Accordingly, Cluster 5 was selected as the potential bioactive candidate pose for the molecular dynamics study (Fig. S3).


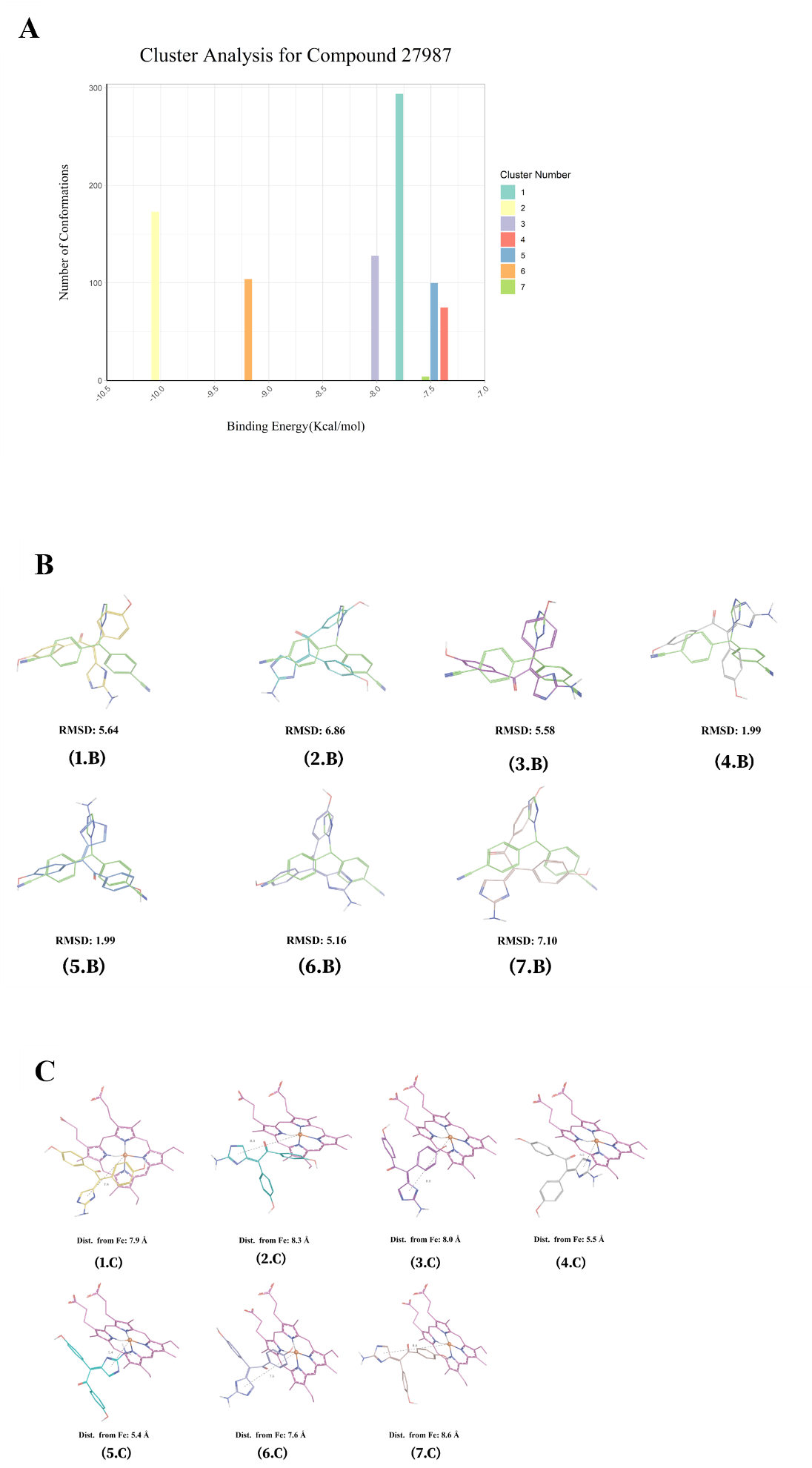


Fig. S3. (A) the outcomes of cluster analysis for CMNPD27987, (B) the RMSD values of the azaheterocyclic ring in each cluster representative when compared to letrozole, and (C) the distances between the azaheterocyclic ring and the Fe of the heme group.

For CMNPD11121, seven clusters were identified as well. Cluster 3 exhibited the best fit to the reference binding pose of letrozole with the second highest number of conformers and mean binding energy at 102 and -9.79 Kcal/mol respectively. Its azaheterocyclic ring showed an RMSD of 1.85 Å compared to the reference ring of letrozole, and the distance from the Fe atom at the heme moiety was 4.6 Å (Fig. S4).


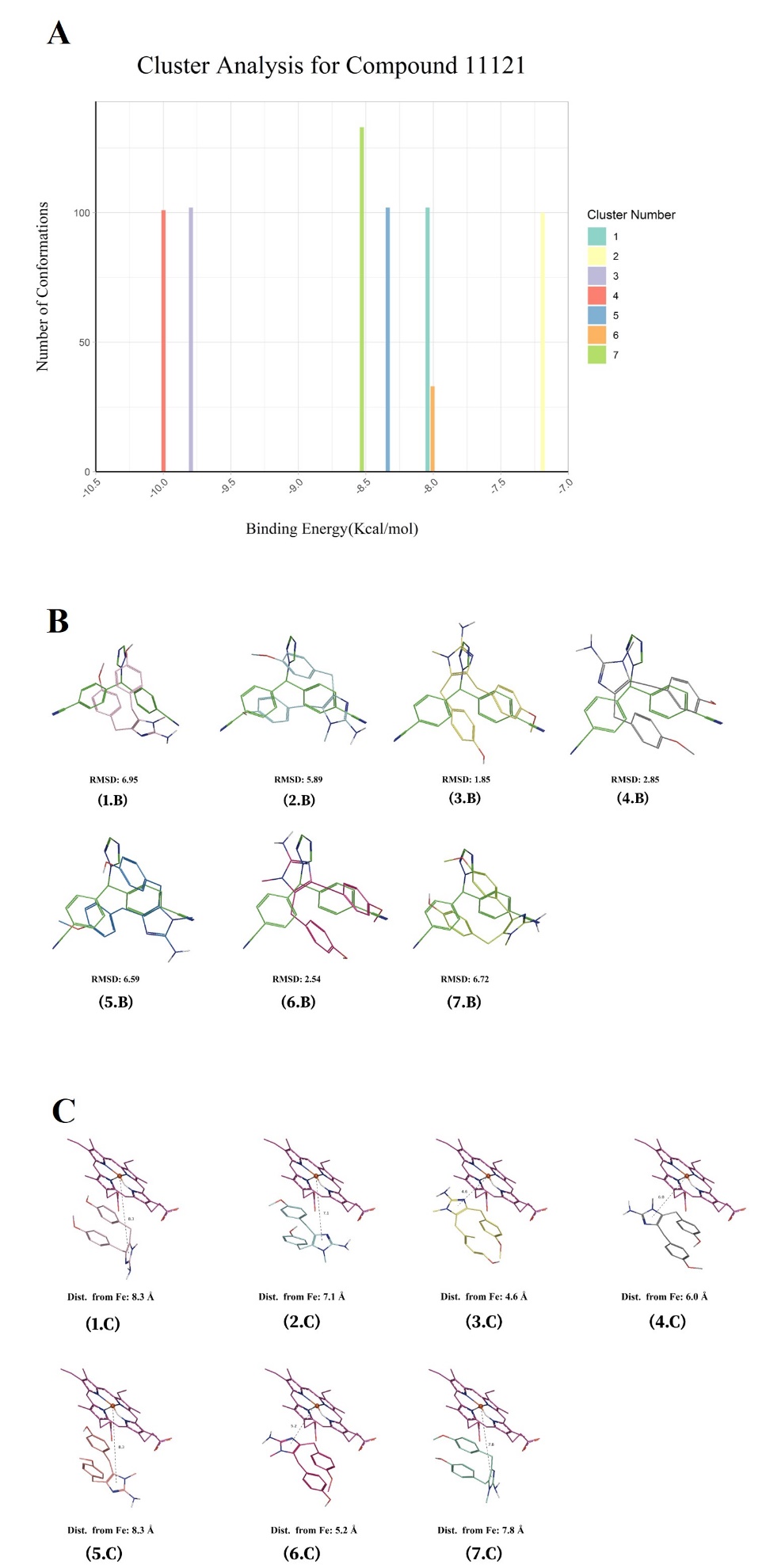


Fig. S4. The figure displays (A) the results of the cluster analysis for CMNPD11121, (B) the RMSD values of the azaheterocyclic ring in each cluster representative in comparison to letrozole, and (C) the distances between the azaheterocyclic ring and the Fe of the metal center.

CMNPD7905 had 5 potential bioactive candidate poses from its nine identified clusters. Cluster 2 had a total of 101 conformers and a binding energy of -7.3 Kcal/mol. Its azaheterocyclic ring had an RMSD of 2.11 Å when compared to that of letrozole and its distance from the heme center was recorded at 5.6 Å. Cluster 5 demonstrated the best pose overall in terms of binding energy (-8.78 Kcal/mol) and structural similarity to the reference binding pose of letrozole (RMSD of 2.18 Å, distance of 3.9 Å). With an RMSD value of 2.46 Å and distance from heme at 3.5 Å Cluster 7 represented 105 conformers of an average free binding energy of -8.35 Kcal/mol. Cluster 8 had 89 conformers and a binding energy of -6.75 Kcal/mol. Its RMSD was recorded at 1.99 Å while the distance from Fe of the iron porphyrin was 4.6 Å. Lastly, Cluster 9 had a total of 16 conformers, a free binding energy of -6.66 Kcal/mol, RMSD of 2.08 Å, and a distance of 5.4 Å (Fig. S5). Cluster 5 was selected for the subsequent molecular dynamics study.


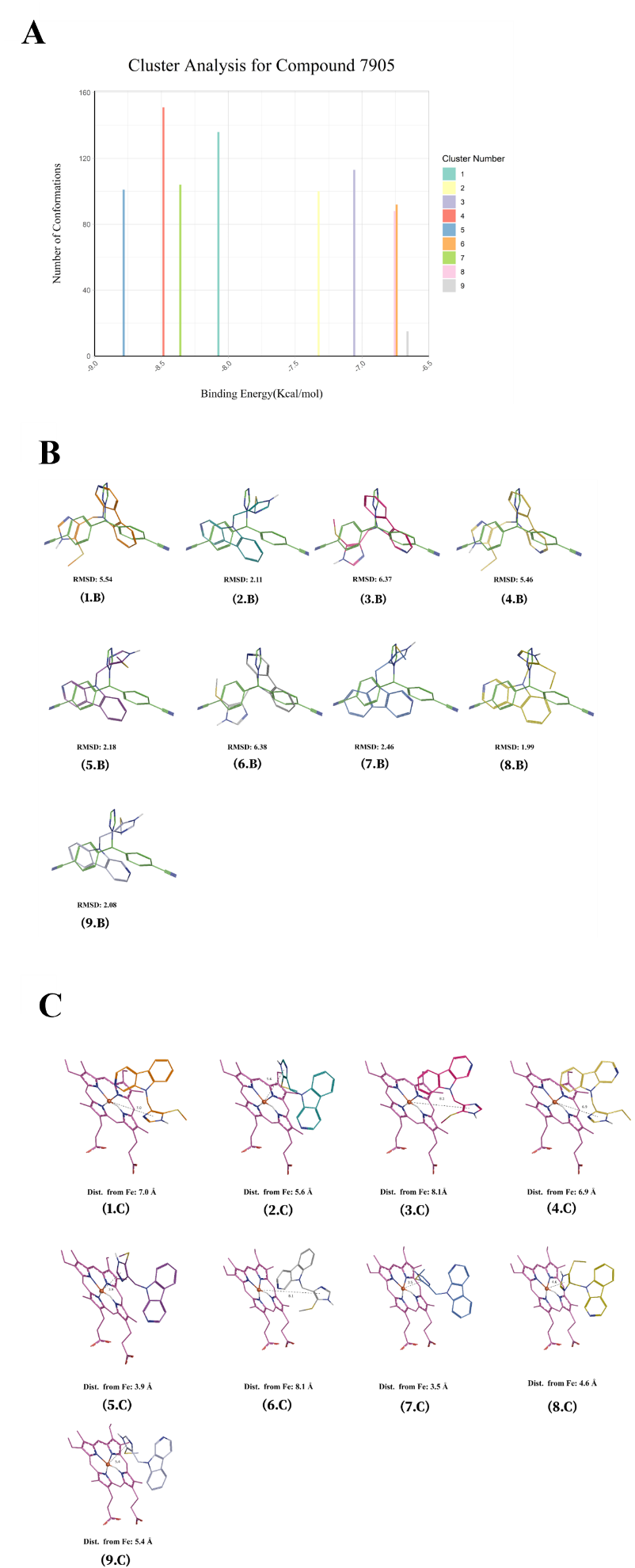


Fig. S5. The figure showcases (A) the outcomes of the cluster analysis for CMNPD7905, (B) the RMSD values of the azaheterocyclic ring in each cluster representative in comparison to letrozole, and (C) the distances between the azaheterocyclic ring and the Fe of the metal center.

Lastly, CMNPD7907 had a total of 11 clusters identified. Two clusters exhibited similarity to the reference binding pose of letrozole. Cluster 2, with a total of 112 conformers, the second highest and -7.70 Kcal/mol free binding energy, had an RMSD value of 2.28 Å when its azaheterocylic ring was compared to that of the reference pose. Additionally, the distance between the Fe atom at the metal coordination center of the binding site and the ring was recorded at 3.8 Å. The second cluster, Cluster 1, had a slightly better distance of 3.6 Å but showed a lower number of conformers, binding energy, and RMSD at values of 101, -7.64 Kcal/mol, and 2.45 Å respectively (Fig. S6). A representative pose from Cluster 2 was selected for the molecular dynamics analysis.


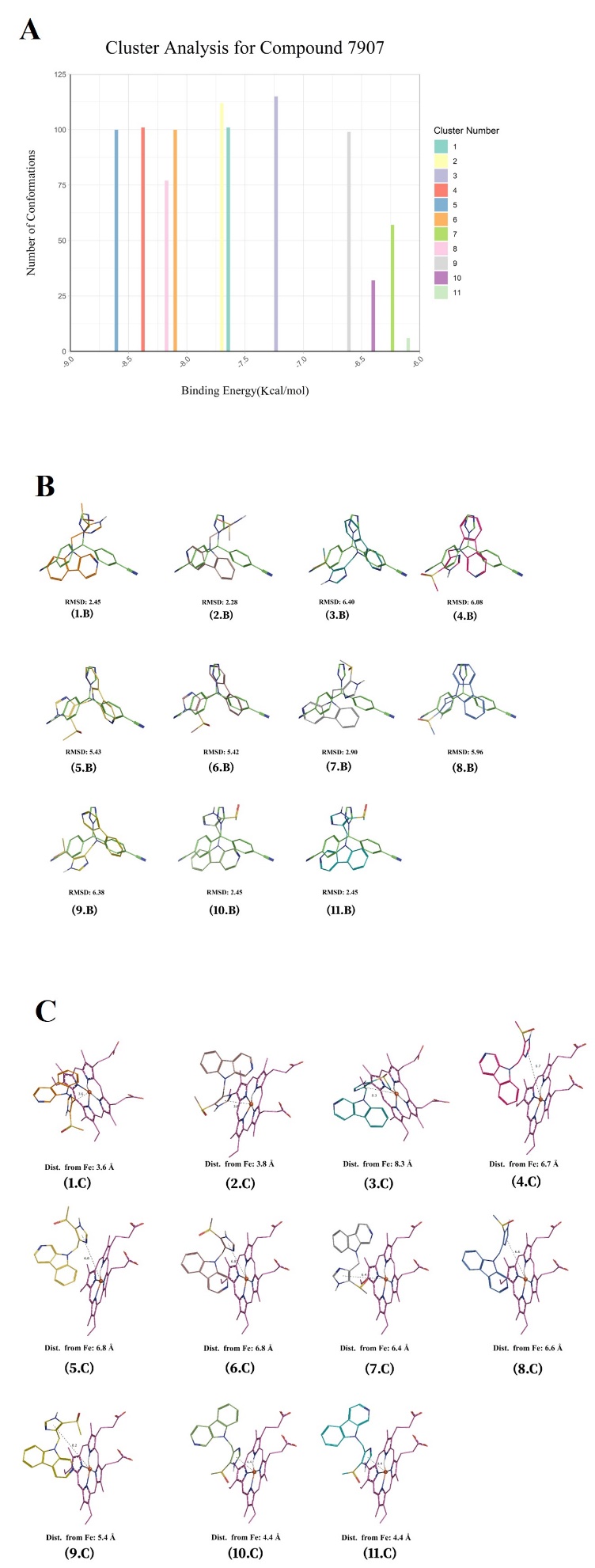


Fig. S6. (A) the results of the cluster analysis for CMNPD7907, (B) the RMSD values of the azaheterocyclic ring in each cluster representative compared to letrozole, and (C) the distances between the azaheterocyclic ring and the Fe of the metal center.

**Summary of protein-ligand interaction profiles**

A ligand-protein interaction analysis was conducted to assess different binding interactions between the functional groups of each of our candidate compounds (CMNPD 27987 11121, 7907, and 7907), in addition to two reference drugs (formestane and letrozole), and the co-crystalized ligand (androstenedione) (Table S1).

**Table S1 The protein-ligand interaction analysis results for lead candidates**

| **Ligand Name** | **Hydrogen Bond Interaction** | **Carbon Hydrogen Interaction** | **Hydrophobic Interaction** | **Van der Waals Interactions** | **Other Interactions** |
| --- | --- | --- | --- | --- | --- |
| Androstenedione | Arg115 (2.76 Å), Met374 (1.89 Å). | Arg115 (3.40 Å), Ala306 (3.26 Å), Val373 (3.92 Å), Met374 (3.62 Å). | Hem600 (3.96 Å), Trp224 (4.96 Å). | Phe221, Leu477, Phe134, Leu372, Val373, Val370, Ile133, Thr310, Ile305, Asp309. | - |
| Formestane | Arg115 (2.64 Å), Met374 (1.82 Å), Asp309 (2.55 Å). | Arg115 (3.29 Å), Ala306 (3.07 Å), Val373 (3.94 Å), Met374 (3.49 Å). | Hem600 (3.99 Å). | Trp224, Ile305, Asp309, Ser478, Phe221, Thr310, Phe134, Leu372, Val373, Val370, Ile133. | - |
| Letrozole | Arg115 (2.73 Å), Met374 (1.76 Å), Ser478 (1.93 Å). | Val373 (3.67 Å), Met374 (3.61 Å), Ser478 (3.28 Å). | Hem600 (5.48 Å, Pi-Pi T-shaped), Ile133 (4.30 Å), Ala306 (4.45 Å), Hem600 (5.24 Å, Pi-Alkyl), Ile133 (5.37 Å), Val370 (5.24 Å). | His480, Val369, Ser478, Leu477, Phe134, Val373, Trp224, Phe221, Asp309, Thr310, HOH630, and Val313. | Hem600 (3.59 Å, Metal acceptor) |
| CMPND 27987 | HOH605 (1.96 Å), Met374 (2.69 Å), Ser478 (2.51 Å), Ser478 (2.81 Å), Arg115 (2.96 Å). | Thr310 (3.57 Å), (Val373 3.51 Å), Ser478 (3.62 Å). | Hem600 (4.72 Å), Ile133 (4.27 Å), Ala309 (5.03 Å), Val370 (5.48 Å, Pi-Alkyl), Val370 (5.04 Å, Pi-Alkyl). | Leu372, Arg115, Phe134, Val313, His480, Val369, Phe221, Asp309, Leu477, and Trp224. | - |
| CMPND 11121 | Leu477 (2.10 Å). | Asp309 (3.31 Å). | Leu477 (3.93 Å), Hem600 (3.89 Å, Pi-Pi Stacked), Ile133 (4.80 Å), Ala306 (4.69 Å), Val370 (5.13 Å), Hem600 (5.10 Å Pi-Alkyl), Val370 (4.97 Å). | Glu302, Val373, Arg115, Met374, Phe134, Leu372, Ser478, Phe221, Thr310, Trp224, Ile305. | - |
| CMPND 7905 | Ala306 (2.31 Å). | - | Thr310 (3.83 Å), Ala306 (3.71 Å), Ile133 (4.21 Å), Ile305 (4.39 Å), Trp224 (4.97 Å), Val370 (5.15 Å, Pi-Alkyl), Ala306 (4.63 Å) Val370 (4.94 Å, Pi-Alkyl), Leu477 (4.66 Å) | Phe134, Arg115, Met374, Val373, Leu372, Ser478, Phe221. | Asp309 (3.30 Å, Sulfur-X), Trp224 (4.94 Å, Pi-Sulfur) |
| CMPND 7907 | - | - | Thr310 (3.59 Å), Leu477(3.87 Å, Pi-Sigma), Hem600 (4.33 Å. Pi-Pi Stacked), Hem600 (4.51 Å. Pi-Pi Stacked), LEU477 (5.42 Å, Pi-Alkyl), Val370, 5.32304 Å) | Val373, Arg115, Ile133, Ala306, Trp224, Ile305, Asp309, Phe221, Ser478, Leu372, Phe134, Met374. | Hem600 (3.40 Å, Electrostatic) |

**References**
1. Raschka S. BioPandas: Working with molecular structures in pandas DataFrames. The Journal of Open Source Software [Internet]. 2017 [cited 2023 Dec 31];2(14):279. Available from: http://joss.theoj.org/papers/10.21105/joss.00279
